# Multi-attribute decision making based on VIKOR with probabilistic linguistic term sets: An application to the risk evaluation of foreign direct investment

**DOI:** 10.1371/journal.pone.0294758

**Published:** 2024-03-01

**Authors:** Xinxin Xu, Yixin Zhang, Zeshui Xu, Huchang Liao, Zhibin Tong

**Affiliations:** 1 Business School, Chengdu University, Chengdu, Sichuan, China; 2 Business School, Sichuan University, Chengdu, Sichuan, China; Gonbad Kavous University, ISLAMIC REPUBLIC OF IRAN

## Abstract

The multiple global environments have triggered changes in the international environment, leading to a sharp decline of foreign direct investment (FDI) compared to pre-pandemic level. To evaluate the investment risk of FDI and make optimal investment decision becomes the most important issue for investors. This paper focuses on the evaluation of investment risk for FDI. First, an index system for risk evaluation of FDI is constructed. Then, we introduce the probabilistic linguistic entropy and cross entropy measures, based on which, a programming model is developed to identify the objective attribute weights. A composite weight derivation method, which takes both the objective attribute weights and the subjective attribute weights into account, is further introduced. In view of attributes’ uncertainty and fuzziness and the conflicting characteristics of some attributes, the VIKOR (the Serbian name: *VlseKriterijumska Optimizacija I Kompromisno Resenje*, means multi-criteria optimization and compromise solution) method is used to evaluate the risk of FDI under the probabilistic linguistic environment. Furthermore, a case study is presented to illustrate the proposed method. The comparative analysis and some further discussions verify the validity of the proposed method for the FDI risk evaluation.

## 1. Introduction

FDI refers to the flows of international direct investment from a country, i.e., investment made by an investor who directly organizes and operates a business in a foreign country. Due to booming merger and acquisition (M&A) markets and rapid growth in international project finance, FDI flows in 2021 increased 64 per cent from the level during the first year of the COVID-19 pandemic to $1.58 trillion. The increase in FDI flows to developed economies (+134 per cent) accounted for most of the global growth, and FDI flows to developing countries reached to $837 billion, increased by 30 per cent to 2020. While the global environment for international investment changed dramatically in 2022 with the onset of the war in Ukraine, food and energy prices, financial turmoil, and debt pressures [1]. The Global Investment Trends Monitoring Report shows that in 2022 the value of M&A sales in the United States fell by 53 per cent, and the number of new greenfield project announcements in China declined by 31 per cent, ASEAN economies reported sharply lower cross-border M&A sales with -74 per cent [2]. The multiple global environments significantly affect investor uncertainty and risk aversity and put significant downward pressure on global FDI. In this situation, evaluating the risk of FDI under multiple crises and making optimal investment decision become extremely important for international investors. It is an important factor that the investors should consider when making investment decision. Understanding and evaluating potential risk can help investors avoid financial losses and protect investor’s assets. Evaluating investment risks can also enhance the accuracy of investment decisions and reduces the chances of investors making impulsive or unwise decisions due to a lack of risk awareness. This paper proposes a decision framework for FDI risk evaluation which can help the investors to evaluate the investment risk to host countries and make reasonable investment decision.

FDI risk evaluation can be seen as a multi-attribute decision making (MADM) problem, which aims to find the best alternative(s) from a set of available alternatives according to the selected attributes [3]. Constructing an index system for FDI risk evaluation is the primary mission. There have been many studies on this issue [4, 5]. Based on the research on the theory and methods of investment risk assessment from the perspective of fuzzy mathematics (See Table 1), they use many kinds of research methods, and lay a solid foundation for the establishment of risk assessment index system in this study.

**Table 1:** Studies about investment risk assessment of FDI.

Researcher(s)	Year	Methodology	Indicator System
Xiuyuan Xu [6]	2020	Deep neural network (DNN)	Basic resources
			Economics and finance
			Political systems
			Environmental protection
			Resources and energy
Bingjie Li [7]	2022	Fuzzy cluster analysis	Politics and policy
			Economy and finance
			Society and culture
			Technological risks
Wei Zhai [8]	2023	G1 entropy method	Economic risk
			Environmental risk
			Social and cultural risk
			Political risk
Jiade Tan et al. [9]	2023	An extended MABAC method	Political stability
			Credit risk
			Law and regulation
			Financial risk
			Infrastructure risk
Qinhua Xu and William Chung [10]	2017	Data envelopment analysis (DEA) model	Environmental dimension
			Social dimension
			Governance dimension

Note: The comparative analysis between this study and others is showed in Section 4.2.

The existing studies construct thorough index systems containing many quantitative indicators based on the crisp data, which may not always be accessible or reliable in practice. Additionally, the FDI risk evaluation is a quantitative analysis of the complex system composed of political, economic, cultural, and other factors of the host country, resulting in complexity and uncertainty. Therefore, it is more appropriate to evaluate the FDI risk under a fuzzy environment. Traditionally, linguistic variables have been used to express qualitative attributes. However, the use of numerical fuzzy numbers may not be suitable for FDI risk evaluation, as it fails to capture aspects that cannot be quantitatively measured [11]. Therefore, the paper proposes constructing a qualitative index system, in which the attributes are expressed by using fuzzy linguistic information, is suitable and necessary. This approach aligns better with the human’s thinking patterns and cognitive processes. Zadeh [12] firstly introduced a fuzzy linguistic approach to model linguistic information. In the fuzzy linguistic approach, the traditional linguistic variable was defined as “*a variable whose values are not numbers but words or sentences in a natural or artificial language*”. The traditional linguistic variable only allows the DM to use one linguistic term to express his/her judgments. However, uncertainty and hesitancy usually exist in practice due to the limitation of the DM’s knowledge and the complexity of decision-making problems. That is, the DM may be hesitant among multiple linguistic terms when he/she evaluates the object being judged [13]. Meanwhile, the DM sometimes has different preference degrees over different linguistic terms. As a new type of linguistic information representation form, the PLTS [14] not only can express the hesitancy of the DM among multiple linguistic terms, but also can present the different preference degrees over the possible linguistic terms. Thus, we focus on the FDI risk evaluation under the probabilistic linguistic environment.

In the FDI risk evaluation process, the DMs in the decision group often come from various specialty fields and each DM is only skilled in some fields. Meanwhile, each DM has unique characteristics in terms of knowledge, skills, experience, and personality. The attribute weight information is usually completely or partly unknown because that the DMs’ knowledge and expertise on the FDI risk evaluation problem is limited. Thus, it is vital to develop a reasonable method to determine attribute weights. Entropy is a parameter used to measure the uncertainty of information. Entropy-based weight method is an effective attribute weight determination method and many investigations have been made [15, 16]. It determines attribute weights based on the decision information and thus is objective. As a parameter used to measure the divergence of information, cross entropy also needs to be considered when we determine the attribute weights. Thus, an objective weight method based on probabilistic linguistic entropy and cross entropy measures is needed for the FDI risk evaluation under probabilistic linguistic environment. However, the entropy and cross entropy weight method ignores the influence of experiential knowledge and comments on decision results. Thus, sometimes, the weights derived by it may not conform to the actual importance degrees of the attributes, even are contrary to actual situations. To deal with this issue, we take both the objective decision information and the subjective experiential knowledge and comments into account and develop a composite weight method.

To evaluate FDI risk, an appropriate evaluation method is required. Several methods have been developed, but they are based on the crisp numbers [17, 18] or fuzzy numbers [19]. To the best of our knowledge, there has been no research conducted on the FDI risk evaluation under a probabilistic linguistic environment. Moreover, existing evaluation index systems in previous studies do not consider the interactions among attribute indicators. As for such a MADM problem for FDI risk evaluation, it is common for certain attributes to conflict with each other. In such a situation, it is often impossible to find a solution that optimizes all attributes simultaneously. To find the best possible solution is urgent for solving this type of MADM problem within the context of PLTSs. The VIKOR method is an effective method for the MADM problems with conflicting attributes. It has been consistently used in a wide range of areas in the last years. It ranks a set of alternatives in the presence of conflicting attributes and obtains the compromise solution to help the DMs to reach a final decision [20]. The compromise solution refers to a feasible solution which is the closest one to the ideal solution, and the compromise refers to an agreement established by mutual concessions [21]. The main advantage of the VIKOR method is that the compromise solution derived by the VIKOR method not only provides a maximum “group utility” for the “majority” but also minimize the “individual regret” for the “opponent” for a MADM problem with conflicting attributes by mutual concessions.

It is noted that there are many popular methods can be applied to evaluate the FDI risk. For example, TOPSIS [14], the MADM techniques with three-way decisions [22–24], EDAS (Evaluation based on Distance from Average Solution) [25], COPRAS (Complex Proportional Assessment) [26], WASPAS (Weighted Aggregates Sum Product Assessment) [27], SECA (Simultaneous Evaluation of Criteria and Alternatives) [28], CODAS (Combinative Distance-based Assessment) [29], SWARA (Stepwise Weight Assessment Ratio Analysis) [30], MEREC (Method based on the Removal Effects of Criteria) [31]. Compared with these methods, the VIKOR is a MCDM technique designed to rank a set of alternatives in the presence of conflicting criteria by proposing a compromise solution. It is effective for the MADM problems with conflicting attributes. In the above discussions, it is showed that some attributes conflict with each other for the FDI risk evaluation issue. Thus, using the VIKOR is a proper way to find a compromise solution. In the future, we will focus on the theoretical research and comparative analysis of the mentioned methods and study the characteristics of VIKOR, EDAS, COPRAS, WASPAS, etc. This helps us better understand the advantages and disadvantages of these approaches. On the other hand, for the FDI risk evaluation issue, we will apply these methods to solve this problem and use case study to analyze and compare the decision results obtained by different methods.

Based on the above analysis, this paper focuses on the FDI risk evaluation with unknown attribute weight information under the probabilistic linguistic environment and aims to develop a decision-making framework for the FDI risk evaluation based on probabilistic linguistic entropy, cross entropy, and VIKOR methods. The motivations and contributions of this paper can be summarized as follows:

1. Considering that the multiple global environments significantly affect investor uncertainty and risk aversity and put significant downward pressure on global FDI, evaluating the risk of FDI is necessary. It helps us to make scientific and optimal investment decisions. Thus, this paper focus on the DFI risk evaluation issue and proposes a decision framework which can help the investors to evaluate the investment risk.
2. Considering that evaluating the DFI risk is a complex problem, constructing the corresponding index system is important. Meanwhile, some aspects of the activities cannot be evaluated by quantitative information because of the qualitative nature of the attributes. Thus, this paper improves the existing risk evaluation index and puts forward a new qualitative evaluation index system.
3. Considering that there exists great complexity and uncertainty for the assessment values of evaluation indexes, the indexes need to be depicted as fuzzy data. We need to obtain the assessment values of indexes with the help of experts. The experts are more inclined to express their judgements in the form of linguistic terms, and PLTS is a new type of linguistic information representation form which is more effective than other fuzzy linguistic information, Thus, this paper evaluate the DFI risk under the probabilistic linguistic environment.
4. Considering that the attribute weight information is usually completely or partly unknown because that the DMs’ knowledge and expertise on the OFDI risk evaluation problem are limited, this paper develop a composite weight method based on the entropy, cross entropy, and the subjective experiential knowledge. The composite weight method takes both the objective decision information and the subjective experiential knowledge and comments into account.
5. Considering that as for the DFI risk evaluation issue, it is common that some indicators some attributes conflict with each other, it is urgent to find the best possible solution. Thus, this paper applies the VIKOR method to solve this issue and proposed the PL-VIKOR method to evaluate the DFI risk.

The remainder of the paper is structured as follows: Section 2 provides an overview of the basic concepts related to PLTS and the VIKOR method, and an index system is built based on the connotations of the FDI risk. Then, we present the probabilistic linguistic entropy and cross entropy measures and develop a programming model to identify the objective attribute weights. A composite weight derivation method is further presented to determine final attribute weights. After that, an extended VIKOR method based on the PLTS is introduced and the detailed algorithm steps for evaluating the FDI risk are presented. In Section 3, a case study is presented to illustrate the proposed method. Section 4 discusses the case results. Section 5 makes a comparative analysis to validate our approach. Finally, Section 6 ends the paper with some conclusions.

## 2. Preliminaries and methodology

### 2.1. Some concepts related to PLTSs

Given an additive linguistic evaluation scale *S* = {s_-*τ*_,⋯,*s*_-1_,*s*_0_,*s*_1_,⋯,s_*τ*_} (*τ* is a positive integer), the PLTS is defined as [14]:

$$L(p)=\left\{L^{\left(l\right)}\left(p^{\left(l\right)}\right)|L^{\left(l\right)}\in S;p^{\left(l\right)}\geq 0;l=1,2,\cdots ,\#L(p);\sum_{l=1}^{\#L(p)}p^{\left(l\right)}\leq 1\right\}$$ (1)

where *L*^(*l*)^ is the linguistic term, *p*^(*l*)^ is the corresponding probability of *L*^(*l*)^, and #*L*(*p*) is the number of linguistic terms in *L*(*p*). The normalized PLTS (NPLTS) is denoted as [14]: $L{\left(p\right)}^{N}=\left\{L^{N(l)}\left(p^{N(l)}\right)|L^{N(l)}=L^{\left(l\right)}\in S;p^{N(l)}\geq 0;\sum_{l=1}^{\#L(p)}p^{N(l)}=1\right\}$, where $p^{N(l)}=\frac{p^{\left(l\right)}}{\sum_{l=1}^{\#L(p)}p^{\left(l\right)}}$.

Because that the PLTSs usually have different numbers of elements, the extension rule [9] needs to be conducted to prevent the trouble in operation. For two PLTSs $L{\left(p\right)}_{1}=\left\{L_{1}^{\left(l\right)}\left(p_{1}^{\left(l\right)}\right)|l=\right.\left.1,2,\cdots ,\#L{\left(p\right)}_{1}\right\}$ and $L{\left(p\right)}_{2}=\left\{L_{1}^{\left(l\right)}\left(p_{1}^{\left(l\right)}\right)|l=\right.\left.1,2,\cdots ,\#L{\left(p\right)}_{2}\right\}$ (#*L*(*p*)_1_ ≠ #*L*(*p*)_2_), if #*L*(*p*)_1_ > #*L*(*p*)_2_, then we will add #*L*(*p*)_1_ - #*L*(*p*)_2_ linguistic terms to *L*(*p*)_2_ (the added linguistic terms are the smallest one in *L*(*p*)_2_, and the probabilities of all the added linguistic terms are equal to zero); if #*L*(*p*)_1_ > #*L*(*p*)_2_, then #*L*(*p*)_2_ − #*L*(*p*)_1_ linguistic terms are added to *L*(*p*)_1_ (the added linguistic terms are the smallest one in *L*(*p*)_1_, and the probabilities of all the added linguistic terms are equal to zero).

Moreover, the elements in each PLTS can change their positions at random. To determine the operational results of PLTSs straightforwardly, the ranking rule is introduced [32]: given the PLTS *L*(*p*) = {*L*^(*l*)^(*p*^(*l*)^)|*l* = 1,2,⋯,#*L(p)*}, we have: (1) for the elements in *L*(*p*), which have different values of *γ*^(*l*)^*p*^(*l*)^, the elements are arranged based on the values of *γ*^(*l*)^*p*^(*l*)^; (2) for the elements in *L*(*p*), which have equal values of *γ*^(*l*)^*p*^(*l*)^, a) if the subscripts *γ*^(*l*)^ of the linguistic term *L*^(*l*)^ are unequal, then these elements are arranged according to the values of *γ*^(*l*)^; b) if the subscripts *γ*^(*l*)^ are equal, then these elements are arranged according to the values of *p*^(*l*)^.

Given the PLTS *L*(*p*) = {*L*^(*l*)^(*p*^(*l*)^)|*l* = 1,2,⋯,#*L*(*p*)}, the score of *L*(*p*) is $\mu (L(p))=s_{\bar{\alpha }}$, and the deviation degree of *L*(*p*) is $v(L(p))=\left(\frac{1}{\sum_{l=1}^{\#L(p)}p^{\left(l\right)}}\right)\sqrt{\sum_{l=1}^{\#L(p)}{\left(p^{\left(l\right)}\left(\gamma^{\left(l\right)}-\bar{\alpha }\right)\right)}^{2}}$ ($\bar{\alpha }=\left(\frac{1}{\sum_{l=1}^{\#L(p)}p^{\left(l\right)}}\right)\sum_{l=1}^{\#L(p)}\gamma^{\left(l\right)}p^{\left(l\right)}$). Then, the PLTSs *L*(*p*)_1_ and *L*(*p*)_2_ are compared based on the scores and the deviation degrees [14]: if *μ*(*L*(*p*)_1_) > *μ*(*L*(*p*)_2_), then *L*(*p*)_1_ ≻ (*L*(*p*)_2_; if *μ*(*L*(*p*)_1_) < *μ*(*L*(*p*)_2_), then *L*(*p*)_1_ ≺ *L*(*p*)_2_; if *μ*(*L*(*p*)_1_) = *μ*(*L*(*p*)_2_), then the further comparison is conducted: if *v*(*L*(*p*)_1_) > *v*(*L*(*p*)_2_), then *L*(*p*)_1_ ≺ *L*(*p*)_2_; if *v*(*L*(*p*)_1_) = *v*(*L*(*p*)_2_), then *L*(*p*)_1_ ~ *L*(*p*)_2_; if *v*(*L*(*p*)_1_) < *v*(*L*(*p*)_2_), then *L*(*p*)_1_ ≻ *L*(*p*)_2_.

For the PLTS *L*(*p*) = {*L*^(*l*)^(*p*^(*l*)^)|*l* = 1,2,⋯,#*L*(*p*)}, the negation operation for *L*(*p*) is defined as $neg(L(p))=\left\{{\hat{L}}^{\left(l\right)}\left(p^{\left(l\right)}\right)|{\hat{L}}^{\left(l\right)}=neg\left(L^{\left(l\right)}\right)=neg\left(s_{\gamma^{\left(l\right)}}\right);l=1,2,\cdots ,\#L(p)\right\}$, where $neg\left(s_{\gamma^{\left(l\right)}}\right)$ is the negation operation for linguistic terms, defined as *neg*(*s*_*α*_) = *s*_-*α*_ with *neg*(*s*_0_) = *s*_0_.

### 2.2. VIKOR

The VIKOR, proposed by Opricovic [33], is a classical MADM method. The VIKOR method introduces the ranking indices *S*_*i*_, *R*_*i*_, and *Q*_*i*_ based on the *L_ρ_*−metric, which is an aggregation function in the compromise programming and represents the “closeness” to the “ideal” solution [34, 35]:

$$\left.L_{\rho ,i}={\left(\sum_{j=1}^{m}{\left(w_{j}\frac{g_{j}^{+}-g_{ij}}{g_{j}^{+}-g_{j}^{-}}\right)}^{\rho }\right)}^{\frac{1}{\rho }},\cdot \rho \in 1,\infty \right),\cdot i=1,2,\cdots ,n$$ (2)

where *w*_*j*_ (*j* = 1,2,⋯,*m*) are the weights of the attributes. *g*_*ij*_ reflects the performance of the region *a*_*i*_ concerning the attribute *c*_*j*_, and $g_{j}^{+}=max_{i}g_{ij}$, $g_{j}^{-}=min_{i}g_{ij}$ for benefit attributes. For the cost attributes, we have $g_{j}^{+}=min_{i}g_{ij}$, $g_{j}^{-}=max_{i}g_{ij}$.

The detailed steps of the classical VIKOR method are presented as follows:

1. Find $g_{j}^{+}$ and $g_{j}^{-}$;
2. Determine the values of *S*_*i*_ and *R*_*i*_, where $S_{i}=L_{1,i}=\sum_{j=1}^{m}\left(w_{j}\frac{\left(g_{j}^{+}-g_{ij}\right)}{\left(g_{j}^{+}-g_{j}^{-}\right)}\right)$ and $R_{i}=L_{\infty ,i}=max_{j}\left(w_{j}\frac{\left(g_{j}^{+}-g_{ij}\right)}{\left(g_{j}^{+}-g_{j}^{-}\right)}\right)$.
3. Determine the values of *Q*. $Q_{i}=\theta \left(\frac{\left(S_{i}-S^{-}\right)}{\left(S^{+}-S^{-}\right)}\right)+(1-\theta )\left(\frac{\left(R_{i}-R^{-}\right)}{\left(R^{+}-R^{-}\right)}\right)$ where *S*^+^ = *max*_*i*_ *S*_*i*_, *S*^-^ = *min*_*i*_ *S*_*i*_, *R*^+^ = *max*_*i*_ *R*_*i*_, *R*^-^ = *min*_*i*_ *R*_*i*_.
4. Rank *S*_*i*_, *R*_*i*_, and *Q*_*i*_ in ascending order, respectively. Then we get three ranking lists.
5. Determine the unique compromise solution *a*_*i*_ with the minimum value of *Q*_*i*_. Meanwhile, the following two conditions should be satisfied:
  1. Acceptable advantage: $Q\left(a_{1^{*}}\right)-Q\left(a_{2^{*}}\right)\geq \frac{1}{\left(n-1\right)}$, where $Q\left(a_{1^{*}}\right)$ is the first smallest value of *Q*_*i*_, $Q\left(a_{2^{*}}\right)$ is the second smallest value of *Q*_*i*_.
  2. Acceptable stability in decision making: $a_{1^{*}}$ should also rank the first in the ranking list by *S*_*i*_ and *R*_*i*_.

Sometimes, these two conditions cannot be met simultaneously. If the first condition cannot be satisfied, then the regions $a_{1^{*}},a_{2^{*}},\cdots ,a_{i^{*}}$ are compromise solutions, where $a_{i^{*}}$ is determined by $Q\left(a_{i^{*}}\right)-Q\left(a_{1^{*}}\right)<\frac{1}{\left(n-1\right)}$ for the maximum i^*^ (the position of these regions are “in closeness”). If the second condition is not satisfied, then the regions $a_{1^{*}}$ and $a_{2^{*}}$ are compromise solutions. The VIKOR method not only maximizes the group utility of the “majority”, but also minimizes the individual regret of the “opponent”. It is adept in finding the compromise solutions among many decision options for the MADM problems with conflicting attributes.

### 2.3. Methodology

This section first builds an index system for the risk evaluation of FDI. Then, a composite attribute weight derivation method based on probabilistic linguistic entropy and cross entropy measures is introduced and the probabilistic linguistic VIKOR (PL-VIKOR) method for evaluating the WHH is presented.

#### 2.3.1. Index system for the risk evaluation of FDI

In this paper, the risk evaluation of FDI (REO) system comprises five components: national political risk, economic risk, cultural risk, institutional risk, and disaster risk [36]. These five components are not only relatively independent, but also interconnected and mutually influencing.

National political risk measures the damage or loss of investment property, rights and interests that foreign investors may encounter during cross-border investment due to political changes in the host country. National political risk, which is often the outcome of human-made incidents in the host country and presents the most significant threat to foreign investors.

National political risk is comprised three subsystems: regime stability risk, encroachment expropriation risk, and risk of varying interventions. As a subsystem, national political risk exhibits multiple properties. Firstly, national political risk possesses the characteristic of differential intervention. This refers to the implementation of stricter policies by host countries on FDI enterprises. Secondly, national political risk encompasses the phenomenon of encroachment expropriation. Although public and direct expropriation risks have generally decreased in recent years, new political risks such as encroachment expropriation have become increasingly prominent. In some cases, host governments require foreign investors to gradually transfer their shares to the host government or nationals over a specified period reaching a majority ownership stake of over 51% or even full ownership of 100% as per the contractual agreement. Finally, the risk of war riots such as war and civil unrest in the host country, as well as the risk of regime stability caused by domestic ethnic conflicts and deterioration of law and order, are macro-level political risks. These risks are often beyond the control of individual and can have a universal negative impact on all businesses operating in the host country, including foreign investments [36].

The economic risk system comprises two subsystems: exchange rate change risk and exchange risk. Exchange rate changes risk refers to the potential increase in liabilities and expenditures, as well as the decrease in assets and income, resulting from fluctuations in foreign exchange rates during the production and business activities of multinational companies. Chinese enterprises involved in overseas investments, often engage in international transactions involving significant amounts of foreign currency. They may also have foreign currency-denominated claims and debts. As a result, they face exchange rate risk, which arises from the changes in the value of their assets and liabilities due to fluctuations in exchange rates. Exchange risk can be categorized into two types, forbidden exchange risk and transfer risk. Forbidden exchange refers to the possibility that an investor may encounter difficulties in converting monetary assets, such as the original investment proceeds or other legally earned income, from the local currency to their home currency or another currency. Transfer risk, on the other hand, pertains to the risk that an investor may face obstacles in transferring monetary assets, including the original investment proceeds or other legally earned income, out of the host country.

The cultural risk subsystem primarily encompasses the risk of cultural conflicts. The risk of clashes between different countries, which is often disregarded during the FDI process, can significantly impact the production and operations of multinational enterprises in extensive and profound ways. Although foreign investment by a country’s enterprises primarily involves the international movement of capital, it also unavoidably entails the exchange, integration and clash of diverse cultures. It is not uncommon for international investment endeavors to encounter obstacles or even fail because of differing backgrounds [36].

The primary risk associated with institutional risk is policy and regulation risk. The policies and regulations implemented by the host country regarding foreign investment have a significant and far-reaching impact on international investment activities. These policies and regulations serve as one of the major of FDI risk for Chinese enterprises. In many developing nations, the challenges of insufficient capital and outdated technology are often addressed by implementing incentive policies to attract foreign investment and technology transfer. However, to safeguard the domestic industry and preserve limited economic resources, various policy and legislative measures are employed to restrict foreign investment to some extent. This is particularly important in countries with weakened economic foundations.

Disaster risk encompasses the potential hazards arising from uncontrollable factors such as natural disasters and public health emergencies. Varied capacities in disaster prevention and post-disaster management exist among different countries, influenced by factors like national economic strength and government execution capabilities. Hence, the exposure of international investors to disaster risks is contingent upon the host country’s geographical characteristics as well as its preparedness and resilience in terms of prevention and response measures.

**Fig 1:**
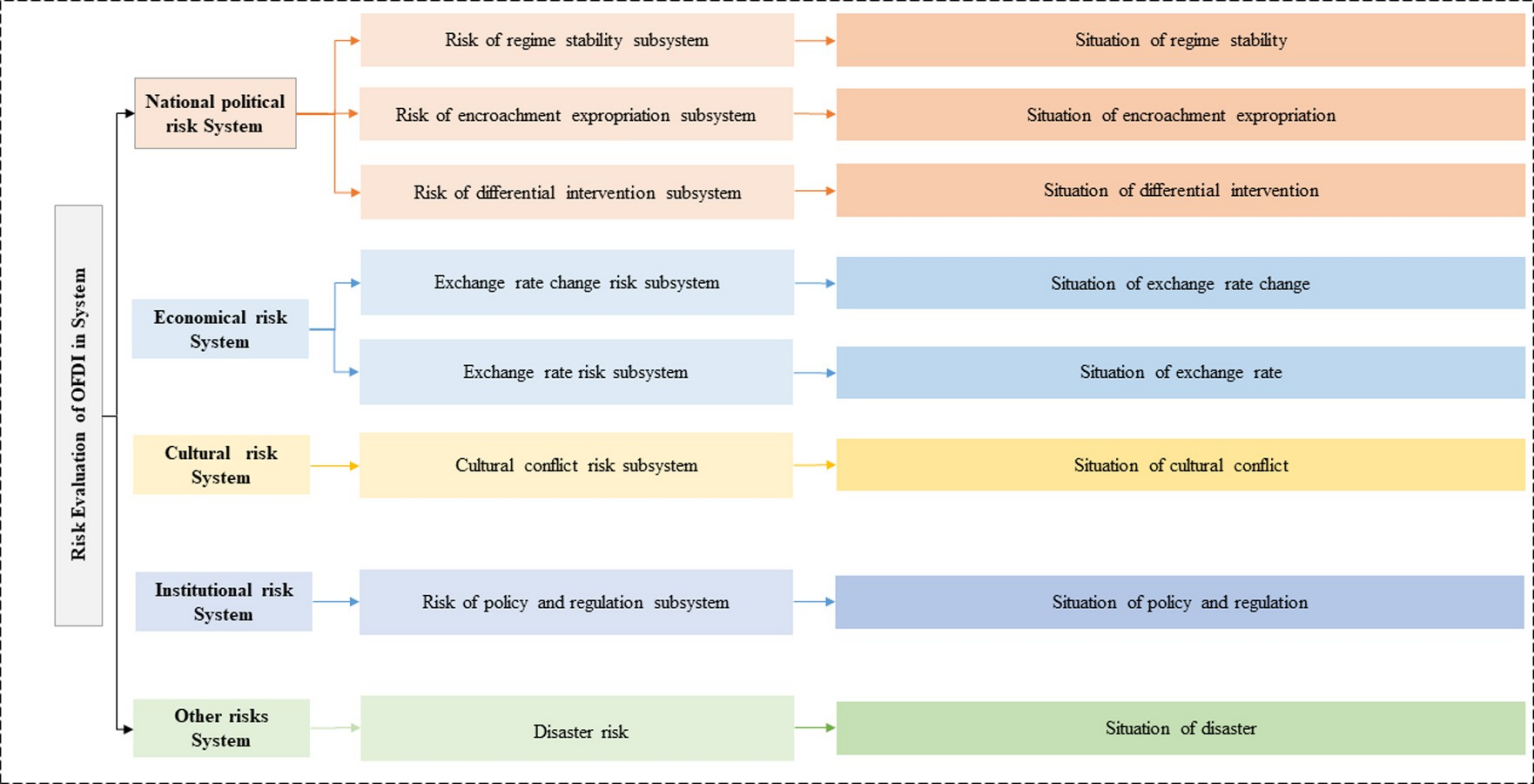
The evaluation index system.

Based on the above analysis, this paper constructs an index system with multiple attribute indicators, as shown in Fig 1. Because the five subsystems and fight specific indicators of the risk evaluation system are all chosen based on the related theories and references, the evaluation index system is not only suitable for China but also for other countries.

We can see that for such a qualitive evaluation index system, it is necessary to obtain the assessment values with the help of experts. To depict these qualitive attributes effectively, fuzzy information is suitable and necessary. Fuzzy linguistic information is more inclined with the human’s thinking habit and cognitive process; thus, experts usually express their assessment values as linguistic information. There are different forms of linguistic information. PLTS is more effective than other linguistic forms because it not only can express the hesitancy of the DM among multiple linguistic terms, but also can present the different preference degrees over the possible linguistic terms. It can retain more of the original decision information of experts. In the actual decision-making process, based on the given linguistic evaluation scale, experts can provide their assessment values of all the indicators as PLTSs. The detailed data obtaining process is explained in the case study.

#### 2.3.2. Entropy and cross entropy-based weight derivation method

Entropy measure in the fuzzy set theory aims to quantify the uncertainty associated to a fuzzy set [34]. It has been extended to different types of fuzzy sets. Liu et al. [16] studied the probabilistic linguistic entropy and defined the fuzzy entropy of the PLTSs as follows:

#### Definition 1 [20]

Let *L*(*p*) = {*L*^(*l*)^(*p*^(*l*)^)|*l* = 1,2,⋯,#*L*(*p*)} be a NPLTS based on the linguistic evaluation scale *S* = {*s*_0_,*s*_1_,⋯,*s_ℓ_*} (*ℓ* is a positive integer). The probabilistic linguistic fuzzy entropy is defined as:

$$FE(L(p))=\sum_{l=1}^{\#L(p)}p^{\left(l\right)}E_{F}\left(\alpha^{\prime (l)}\right)$$ (3)

where *L*(*p*) = {*L*^(*l*)^(*p*^(*l*)^)|*l* = 1,2,⋯,#*L*(*p*)} is a NPLTS based on the linguistic evaluation scale *S* = {*s*_0_,*s*_1_,⋯,*s_ℓ_*} (*ℓ* is a positive integer), $\alpha^{\prime (l)}=\frac{\gamma^{\left(l\right)}}{l}$ and *E*_*F*_(*α*^’(*l*)^) denotes the fuzzy entropy of the hesitant fuzzy sets in Ref. [37]. The formula to measure the probabilistic linguistic fuzzy entropy [11] is $E(L(p))=-\left(\frac{1}{\ln2}\right)\sum_{l=1}^{\#L(p)}p^{\left(l\right)}\left[\alpha^{\prime (l)}\;ln\;\alpha^{\prime (l)}+\left(1-\alpha^{\prime (l)}\right)ln\left(1-\alpha^{\prime (l)}\right)\right]$.

In this paper, the PLTSs are based on the symmetric linguistic evaluation scale *S* = {*s*_*-τ*_,⋯,*s*_-1,_*s*_0,_*s*_1_,⋯,*s*_τ_}, rather than *S* = {*s*_0,_*s*_1_,⋯,*s_ℓ_*}. A new formula of *α*^’(*l*)^ is deduced: $\alpha^{\left(l\right)}=\frac{\left(\gamma^{\left(l\right)}+\tau \right)}{2\tau }$ (*α*^(*l*)^ ∈[0,1]). Naturally, the fuzzy entropy of the PLTSs based on the symmetric linguistic evaluation scale *S* = {*s*_*-τ*_,⋯,*s*_-1,_*s*_0,_*s*_1_,⋯,*s*_τ_} is $FE(L(p))=\sum_{l=1}^{\#L(p)}p^{\left(l\right)}E_{F}\left(\alpha^{\left(l\right)}\right)$, and the calculation formula is

$$FE(L(p))=-\frac{1}{ln\;2}\sum_{l=1}^{\#L(p)}p^{\left(l\right)}\left[\alpha^{\left(l\right)}\;ln\;\alpha^{\left(l\right)}+\left(1-\alpha^{\left(l\right)}\right)ln\left(1-\alpha^{\left(l\right)}\right)\right]$$ (4)

where $\alpha^{\left(l\right)}=\frac{\left(\gamma^{\left(l\right)}+\tau \right)}{2\tau }$. It is noted that different expressions of *E*_*F*_(*α*_*l*_) will derive different expressions of *FE*. This paper does not focus on the entropy measure and thus the fuzzy entropy measure introduced by Liu et al. [16] is directly used.

In addition, cross entropy is an important concept to measure the divergence of information. We define the probabilistic linguistic cross entropy as:

#### Definition 1

Let $L{\left(p\right)}_{1}=\left\{L_{1}^{\left(l\right)}\left(p_{1}^{\left(l\right)}\right)|l=1,2,\cdots ,\#L{\left(p\right)}_{1}\right\}$ and $L{\left(p\right)}_{2}=\left\{L_{2}^{\left(l\right)}\left(p_{2}^{\left(l\right)}\right)|l=1,2,\cdots ,\#L{\left(p\right)}_{2}\right\}$ be two ordered NPLTSs based on the linguistic evaluation scale *S* = {*s*_*-τ*_,⋯,*s*_-1,_*s*_0,_*s*_1_,⋯,*s*_τ_}, the cross entropy of *L*(*p*)_1_ and *L*(*p*)_2_ is:

$$CE\left(L{\left(p\right)}_{1},L{\left(p\right)}_{2}\right)=\sum_{l=1}^{\#L}p_{1}^{\left(l\right)}E_{c}\left(\alpha^{\left(l\right)},\beta^{\left(l\right)}\right)$$ (5)

where $\alpha^{\left(l\right)}=\frac{\left(\gamma_{1}^{\left(l\right)}+\tau \right)}{2\tau }$, $\beta^{\left(l\right)}=\frac{\left(\gamma_{2}^{\left(l\right)}+\tau \right)}{2\tau }$, #*L*(*p*)_1_ = #*L*(*p*)_2_ = #*L* (otherwise, the extension rules in Section 2.1 is used to make #*L*(*p*)_1_ = #*L*(*p*)_2_)), and *E*_*c*_ denotes the cross entropy of the hesitant fuzzy elements (HFEs) [38], which is defined as follows:

#### Definition 2 [39]

Let *α* = {*α*_*l*_|*l* = 1,⋯,*L*} and *β* = {*β*_*l*_|*l* = 1,⋯,*L*} be two HFEs (*α*_*l*_ and *β*_*l*_ denote the possible membership degrees in *α* and *β*, respectively, and *α*_*l*_,*β*_*l*_∈[0,1]), then the cross entropy *E*_*c*_(*α*,*β*) of *α* and *β* should satisfy the following conditions: (1) *E*_*c*_(*α*,*β*) ≥ 0;

(2) *E*_*c*_(*α*,*β*) = 0 iff *α*_σ(*l*)_ = *β*_σ(l)_, *l* = 1,2,⋯,*L*, and the formula of *E*_*c*_(*α*,*β*) is

$$E_{c}(\alpha ,\beta )=\frac{1}{L}\sum_{l=1}^{L}E_{c}\left(\alpha_{l},\beta_{l}\right)$$

$$=\frac{1}{L\Theta }\sum_{l=1}^{L}\left(\frac{\left(1+q\alpha_{\sigma (l)}\right)ln\left(1+q\alpha_{\sigma (l)}\right)+\left(1+q\beta_{\sigma (l)}\right)ln\left(1+q\beta_{\sigma (l)}\right)}{2}\right.$$

$$-\frac{2+q\alpha_{\sigma (l)}+q\beta_{\sigma (l)}}{2}ln\frac{2+q\alpha_{\sigma (l)}+q\beta_{\sigma (l)}}{2}$$

$$+\frac{\left(1+q\left(1-\alpha_{\sigma (L-l+1)}\right)\right)ln\left(1+q\left(1-\alpha_{\sigma (L-l+1)}\right)\right)+\left(1+q\left(1-\beta_{\sigma (L-l+1)}\right)\right)ln\left(1+q\left(1-\beta_{\sigma (L-l+1)}\right)\right)}{2}$$

$$\left.-\frac{2+q\left(1-\alpha_{\sigma (L-l+1)}+1-\beta_{\sigma (L-l+1)}\right)}{2}ln\frac{2+q\left(1-\alpha_{\sigma (L-l+1)}+1-\beta_{\sigma (L-l+1)}\right)}{2}\right)$$ (6)

where Θ=(1+q)ln(1+q)−(2+q)(ln(2+q)−ln2)(q>0).

Then, the probabilistic linguistic cross entropy measure can be represented as:

$$CE\left(L{\left(p\right)}_{1},L{\left(p\right)}_{2}\right)=\sum_{l=1}^{\#L}p_{1}^{\left(l\right)}E_{c}\left(\alpha^{\left(l\right)},\beta^{\left(l\right)}\right)$$

$$=\frac{1}{\Theta }\sum_{l=1}^{\#L}p_{1}^{\left(l\right)}\left(\frac{\left(1+q\alpha^{\sigma (l)}\right)ln\left(1+q\alpha^{\sigma (l)}\right)+\left(1+q\beta^{\sigma (l)}\right)ln\left(1+q\beta^{\sigma (l)}\right)}{2}\right.$$

$$-\frac{2+q\alpha^{\sigma (l)}+q\beta^{\sigma (l)}}{2}ln\frac{2+q\alpha^{\sigma (l)}+q\beta^{\sigma (l)}}{2}$$

$$+\frac{\left(1+q\left(1-\alpha^{\sigma (\#L-l+1)}\right)\right)ln\left(1+q\left(1-\alpha^{\sigma (\#L-l+1)}\right)\right)+\left(1+q\left(1-\beta^{\sigma (\#L-l+1)}\right)\right)ln\left(1+q\left(1-\beta^{\sigma (\#L-l+1)}\right)\right)}{2}$$

$$\left.-\frac{2+q\left(1-\alpha^{\sigma (\#L-l+1)}+1-\beta^{\sigma (\#L-l+1)}\right)}{2}ln\frac{2+q\left(1-\alpha^{\sigma (\#L-l+1)}+1-\beta^{\sigma (\#L-l+1)}\right)}{2}\right)$$ (7)

Especially, when $p_{1}^{\left(l\right)}=\frac{1}{\#L}$ for *l* = 1,2,⋯,#*L*, $CE\left(L{\left(p\right)}_{1},L{\left(p\right)}_{2}\right)=\sum_{l=1}^{\#L}p_{1}^{\left(l\right)}E_{c}\left(\alpha^{\left(l\right)},\beta^{\left(l\right)}\right)=\frac{1}{L}\sum_{l=1}^{L}E_{c}\left(\alpha^{\left(l\right)},\beta^{\left(l\right)}\right)=E_{c}(\alpha ,\beta )$. The cross entropy of HFEs can be regarded as a special case of the cross entropy of PLTSs.

#### Theorem 1

The probabilistic linguistic cross entropy *CE*(*L*(*p*)_1_,*L*(*p*)_2_) also satisfies the conditions of the cross entropy for HFEs in Definition 2:

1. *CE*(*L*(*p*)_1_,*L*(*p*)_2_) ≥ 0;
2. *CE*(*L*(*p*)_1_,*L*(*p*)_2_) = 0 if and only if *α*^(*l*)^ = *β*^(*l*)^, *l* = 1,2,⋯,#*L*.

#### Proof

When we do not consider the probability information in *L*(*p*)_1_ and *L*(*p*)_2_, *L*(*p*)_1_ and *L*(*p*)_2_ are degenerated into HFEs $\alpha =\left\{\alpha^{\left(l\right)}=\frac{\left(\gamma_{1}^{\left(l\right)}+\tau \right)}{2\tau }|l=1,\cdots ,\#L\right\}$ and $\beta =\left\{\beta^{\left(l\right)}=\frac{\left(\gamma_{2}^{\left(l\right)}+\tau \right)}{2\tau }|l=1,\cdots ,\#L\right\}$, respectively.

1. Since $E_{c}(\alpha ,\beta )=\frac{1}{\#L}\sum_{l=1}^{\#L}E_{c}\left(\alpha^{\left(l\right)},\beta^{\left(l\right)}\right)\geq 0$, then we have *E*_*c*_(*α*^(*l*)^,*β*^(*l*)^) ≥ 0. It is obvious that $CE\left(L{\left(p\right)}_{1},L{\left(p\right)}_{2}\right)=\sum_{l=1}^{\#L}p_{1}^{\left(l\right)}E_{c}\left(\alpha^{\left(l\right)},\beta^{\left(l\right)}\right)\geq 0$ because that *E*_*c*_(*α*^(*l*)^,*β*^(*l*)^) ≥ 0 and $p_{1}^{\left(l\right)}\geq 0$.
2. When *α*^(*l*)^ = *β*^(*l*)^ (*l* = 1,⋯,#*L*), it is obvious that $E_{c}(\alpha ,\beta )=\left(\frac{1}{\#L}\right)\sum_{l=1}^{\#L}E_{c}\left(\alpha^{\left(l\right)},\beta^{\left(l\right)}\right)=0$ and thus *E*_*c*_(*α*^(*l*)^,*β*^(*l*)^) = 0. Then we have $E\left(L{\left(p\right)}_{1},L{\left(p\right)}_{2}\right)=\sum_{l=1}^{\#L}p_{1}^{\left(l\right)}E_{c}\left(\alpha^{\left(l\right)},\beta^{\left(l\right)}\right)=0$. That is, *CE*(*L*(*p*)_1_,*L*(*p*)_2_) = 0 if *α*^(*l*)^ = *β*^(*l*)^ (*l* = 1,⋯,#*L*). In turn, when $CE\left(L{\left(p\right)}_{1},L{\left(p\right)}_{2}\right)=\sum_{l=1}^{\#L}p_{1}^{\left(l\right)}E_{c}\left(\alpha^{\left(l\right)},\beta^{\left(l\right)}\right)=0$, $p_{1}^{\left(l\right)}=0$ or *E*_*c*_(*α*^(*l*)^,*β*^(*l*)^) = 0 for *l* = 1,⋯,#*L*. Since it is impossible that $p_{1}^{\left(l\right)}=0$ for *l* = 1,⋯,#*L*, then we have *E*_*c*_(*α*^(*l*)^,*β*^(*l*)^) = 0 for *l* = 1,⋯,#*L*. Thus, *α*^(*l*)^ = *β*^(*l*)^ for *l* = 1,⋯,#*L*. That is, *α*^(*l*)^ = *β*^(*l*)^ (*l* = 1,⋯,#*L*) if *E*_*c*_(*L*(*p*)_1_,*L*(*p*)_2_) = 0.

**Note:** In a similar way, we can prove that *CE*(*L*(*p*)_2_,*L*(*p*)_1_) ≥ 0 and *CE*(*L*(*p*)_2_,*L*(*p*)_1_) = 0 if and only if *β*^(*l*)^ = *α*^(*l*)^, *l* = 1,⋯,#*L*.

#### Definition 3

Let $L{\left(p\right)}_{1}=\left\{L_{1}^{\left(l\right)}\left(p_{1}^{\left(l\right)}\right)|l=1,2,\cdots ,\#L{\left(p\right)}_{1}\right\}$ and $L{\left(p\right)}_{2}=\left\{L_{2}^{\left(l\right)}\left(p_{2}^{\left(l\right)}\right)|l=\right.\left.1,2,\cdots ,\#L{\left(p\right)}_{2}\right\}$ be two ordered NPLTSs based on the linguistic evaluation scale *S* = {*s*_*-τ*_,⋯,*s*_-1,_*s*_0,_*s*_1_,⋯,*s*_τ_}, the symmetric cross entropy of *L*(*p*)_1_ and *L*(*p*)_2_ is:

$$CE_{s}\left(L{\left(p\right)}_{1},L{\left(p\right)}_{2}\right)=\frac{1}{2}\left(CE\left(L{\left(p\right)}_{1},L{\left(p\right)}_{2}\right)+CE\left(L{\left(p\right)}_{2},L{\left(p\right)}_{1}\right)\right)$$

$$=\frac{1}{2}\left(\sum_{l=1}^{\#L}p_{1}^{\left(l\right)}E_{c}\left(\alpha^{\left(l\right)},\beta^{\left(l\right)}\right)+\sum_{l=1}^{\#L}p_{2}^{\left(l\right)}E_{c}\left(\beta^{\left(l\right)},\alpha^{\left(l\right)}\right)\right)$$ (8)

The symmetric probabilistic linguistic cross entropy *CE*_*s*_(*L*(*p*)_1_,*L*(*p*)_2_) can be used to measure the difference between *L*(*p*)_1_ and *L*(*p*)_2_. According to Theorem 1, it is easy to prove that *CE*_*s*_(*L*(*p*)_1_,*L*(*p*)_2_) ≥ 0 and *CE*_*s*_(*L*(*p*)_1_,*L*(*p*)_2_) = 0 if and only if *α*^(*l*)^ = *β*^(*l*)^, *l* = 1,⋯,#*L*.

To determine the objective weights, the symmetric probabilistic linguistic cross entropy is used to measure the deviation degree of the country *a*_*i*_ to all the other countries on the attribute *c*_*j*_ firstly, denoted as:

$$DD_{ij}=\frac{1}{n-1}\sum_{k=1,k\neq i}^{n}CE_{s}\left(L{\left(p\right)}_{ij},L{\left(p\right)}_{kj}\right)$$ (9)

Then, the cross entropy of the attribute *c*_*j*_ is $DD_{j}=\sum_{i=1}^{n}DD_{ij}$, which reflects divergence of all the countries on the attribute *c*_*j*_. If the cross entropy of an attribute is small, then the countries have small deviation under this attribute. That is, the attribute plays a slightly important role and thus should be given a smaller weight.

Besides, we should also take the fuzziness of the decision-making information into consideration when determining the objective weights. The fuzziness degree of the attribute *c*_*j*_ can be formulized by probabilistic linguistic fuzzy entropy as follows:

$$FD_{j}=\sum_{i=1}^{n}\left(FE\left(L{\left(p\right)}_{ij}\right)\right)$$ (10)

The high fuzzy entropy of an attribute indicates that the information has a high fuzziness degree, and the attribute provides little information for decision making. If the attribute has high fuzzy entropy, then a lower weight should be given to it. Otherwise, a higher weight should be given to the attribute.

We can see that *DD*_*j*_ and *FD*_*j*_ respectively depict the information of the attribute *c*_*j*_ from aspects of deviation and fuzziness degrees. Taking both deviation and fuzziness degrees into consideration, we build the following programming model to obtain the optimal attribute weights with the completely unknown weight information:

### Model 1

$$\left\{\begin{array}{l} max\;E(\omega )=\sum_{j=1}^{m}E_{j}\times \omega_{j}=\sum_{j=1}^{m}\sum_{i=1}^{n}\left(\frac{1}{n-1}\;\sum_{k=1,k\neq i}^{n}\left.CE_{s}\left(L{\left(p\right)}_{ij},L{\left(p\right)}_{kj}\right)+\left(1-FE\left(L{\left(p\right)}_{ij}\right)\right)\right)\times \omega_{j}\right.\\ \text{s.t.}\sum_{j=1}^{m}\omega_{j}^{2}=1,\omega_{j}\geq 0,j=1,2,\cdots ,m \end{array}\right.$$

We construct the following Lagrange function:

$$Lag\left(\omega_{j},\zeta \right)=\sum_{j=1}^{m}\sum_{i=1}^{n}\left(\frac{1}{n-1}\sum_{k=1,k\neq i}^{n}CE_{s}\left(L{\left(p\right)}_{ij},L{\left(p\right)}_{kj}\right)+\left(1-FE\left(L{\left(p\right)}_{ij}\right)\right)\right)\omega_{j}+\frac{\zeta }{2}\left(\sum_{j=1}^{m}\omega_{j}^{2}-1\right)$$ (11)

where ζ is the Lagrange multiplier. Then we have

$$\left\{\begin{array}{l} \frac{\partial Lag}{\partial \omega_{j}\sum_{i=1}^{n}\left(\frac{1}{n-1}\sum_{k=1,k\neq i}^{n}CE_{S}\left(L{\left(p\right)}_{ij},L{\left(p\right)}_{kj}\right)+\left(1-FE\left(L{\left(p\right)}_{ij}\right)\right)\right){+}_{j}=}\\ \frac{\partial Lag}{\partial \zeta \sum_{j=1}^{m}\omega_{j}^{2}}\{ \end{array}\right.$$ (12)

By solving the above equation, the formula for determining the attribute weights is deduced as:

$$\omega_{j}^{*}=\frac{\sum_{i=1}^{n}\left(\frac{1}{n-1}\sum_{k=1,k\neq i}^{n}CE_{S}\left(L{\left(p\right)}_{ij},L{\left(p\right)}_{kj}\right)+\left(1-FE\left(L{\left(p\right)}_{ij}\right)\right)\right)}{\sqrt{\sum_{j=1}^{m}{\left(\sum_{i=1}^{n}\left(\frac{1}{n-1}\sum_{k=1,k\neq i}^{n}CE_{S}\left(L{\left(p\right)}_{ij},L{\left(p\right)}_{kj}\right)+\left(1-FE\left(L{\left(p\right)}_{ij}\right)\right)\right)\right)}^{2}}}$$ (13)

The normalized objective attribute weights is

$$\omega_{j}=\frac{\sum_{i=1}^{n}\left(\frac{1}{n-1}\sum_{k=1,k\neq i}^{n}CE_{S}\left(L{\left(p\right)}_{ij},L{\left(p\right)}_{kj}\right)+\left(1-FE\left(L{\left(p\right)}_{ij}\right)\right)\right)}{\sum_{j=1}^{m}\sum_{i=1}^{n}\left(\frac{1}{n-1}\sum_{k=1,k\neq i}^{n}CE_{S}\left(L{\left(p\right)}_{ij},L{\left(p\right)}_{kj}\right)+\left(1-FE\left(L{\left(p\right)}_{ij}\right)\right)\right)}$$ (14)

As stated in Introduction, even if the entropy and cross entropy-based weight method has many advantages, it still does not consider the experiential knowledge and comments. The subjective weights exactly reflect the experiential knowledge and comments. Combining the subjective weights with the entropy and cross entropy overcomes the disadvantages of the entropy and cross entropy based weight method. Thus, this paper proposes a composite weight derivation method, which combines the subjective weight and the objective weight. Let *w*_*j*_ be the weight of the attribute *c*_*j*_, then we have

$$w_{j}=\varepsilon \varpi_{j}+(1-\varepsilon )\omega_{j},0\leq \varepsilon \leq 1$$ (15)

where $\varpi_{j}$ is the weight provided by the experts (i.e., the subjective weight), *ω*_*j*_ is the objective weight, and ε is the parameter determined by the experts. When ε = 0, the composite weight is reduced to the objective weight, while when ε = 1, the composite weight is reduced to the subjective weight.

#### 2.3.3. Risk evaluation of FDI based on the PL-VIKOR method

As for the FDI risk evaluation under the probabilistic linguistic environment, let *A* = {*a*_1_,*a*_2_,⋯,*a*_*n*_} be the finite set of countries, and *C* = {*c*_1_,*c*_2_,⋯,*c*_*m*_} be the set of attributes. *a*_*i*_ denotes the *i*-th country, *i*∈*N* = {1,2,⋯,*n*}. *c*_*j*_ denotes the j-th attribute, *j*∈*M* = {1,2,⋯,*m*}. The attribute weight vector is denoted as *W* = (*w*_1_,*w*_2_,⋯,*w*_*m*_)^T^, where *w*_*j*_ is the weight of the attribute *c*_*j*_, 0 ≤ *w*_*j*_ ≤ 1, and $\sum_{j=1}^{m}w_{j}=1$.

The assessment values of the countries *a*_*i*_ (*i* = 1,2,⋯,*n*) with respect to the attributes *c*_*j*_ (*j* = 1,2,⋯,*m*) are determined by the experts. When an expert is providing his/her assessment value concerning an attribute indicator, he/she is more inclined to express his/her judgment (assessment value) by using linguistic information. Based on the given linguistic evaluation scale *S* = {*s*_*-τ*_,⋯,*s*_-1,_*s*_0,_*s*_1_,⋯,*s*_τ_}, the expert uses the linguistic terms *s*_*α*_ (*α* = -*τ*,⋯,-1,0,1,⋯,*τ*) to express his/her judgments. Moreover, there usually exists great complexity and uncertainty in practical decision-making problems. When evaluating an object, the expert may be hesitant among multiple linguistic terms. Meanwhile, the expert may have different preference degrees over the multiple linguistic terms. The PLTSs can better present the hesitancy of the expert and the expert’s different preference degrees to the possible linguistic terms. For example, when an expert is assessing the situation of regime stability in a host country, he/she thinks that it is “*alittlebad*” (*s*_-1_) or “*bad*” (*s*_-2_). Meanwhile, he/she is 40 percentage sure that it is “*alittlebad*” (*s*_-1_), 40percentage sure that it is “*bad*” (*s*_-2_), and 20 percentage sure that it is neither “*alittlebad*” (*s*_-1_) nor “*bad*” (*s*_-2_), but he/she cannot give the specific linguistic terms. Then, the judgment of the expert can be presented as a PLTS, denoted as *L*(*p*) = {*s*_-2_(0.4),*s*_-1_(0.4)}.

Like the above example in explaining the PLTS, we can obtain all assessment values of different countries over all indicators. All these assessment values consist of the original probabilistic linguistic decision matrix, denoted as:

$$D={\left(L{\left(p\right)}_{ij}\right)}_{n\times m}=\left[\begin{array}{cccc} L{\left(p\right)}_{11} & L{\left(p\right)}_{12} & \cdots & L{\left(p\right)}_{1m}\\ L{\left(p\right)}_{21} & L{\left(p\right)}_{22} & \cdots & L{\left(p\right)}_{2m}\\ \vdots & \vdots & \ddots & \vdots \\ L{\left(p\right)}_{n1} & L{\left(p\right)}_{n2} & \cdots & L{\left(p\right)}_{nm} \end{array}\right]$$ (16)

where *L*(*p*)_*ij*_ denotes the assessment value of the country *a*_*i*_ over the attribute *c*_*j*_.

It should be noted that the collecting process of linguistic information with the PLTSs can also be explained from the perspective of GDM. There are usually more than one expert invited to evaluate the candidate countries. As for the expert group, it is inevitable that the experts may have different opinions/judgments on an attribute indicator. The PLTS can better present the original decision-making information. For example, when the expert group (involved with ten experts) is evaluating the situation of regime stability in a host country, five experts think that the regime stability in this country is “*alittlebad*”, three experts think that the regime stability in this country is “*medium*”, and two experts think that the regime stability in this country is “*bad*”. Then, the judgments of all experts can be integrated into a PLTS, denoted as (*p*) = {*s*_-2_(0.2),*s*_-1_(0.5),*s*_0_(0.3)}.

For any one PLTS $L{\left(p\right)}_{ij}=\left\{L_{ij}^{\left(l\right)}\left(p_{ij}^{\left(l\right)}\right)|l=1,2,\cdots ,\#L{\left(p\right)}_{ij}\right\}$ in the original decision matrix, it is possible that $\sum_{l=1}^{\#L{\left(p\right)}_{ij}}p_{ij}^{\left(l\right)}<1$. The normalization of the PLTSs in the decision matrix can estimate the ignorance of probabilistic information. Thus, the normalization process, presented in Section 2.1, is conducted. Then, the extension rule for PLTSs, presented in Section 2.1, is conducted to normalize the cardinality of a PLTS for the purpose of computation.

What’s more, as for the MADM problem with multiple attribute indicators, there are two types of attributes: benefit attributes and cost attributes. The lager the value of the benefit-attribute indicator is, the better the performance concerning this indicator should be. The smaller the value of the cost-attribute indicator is, the better the performance concerning this indicator should be. In order to get rid of the influence of the attributes with different physical dimensions on the decision results, all attributes need to be transformed into the same compatible measure or non-dimensional as to guarantee the compatibility between all attribute values. This paper transforms the cost-attribute indicators into the benefit-attribute indicators by using the transformation function: *function*(*L*(*p*)) = *neg*((*L*(*p*))^*n*^).

After the above process, the normalized decision matrix with the same length is obtained:

$$ND={\left(L{\left(p\right)}_{ij}^{N}\right)}_{n\times m}=\left[\begin{array}{cccc} L{\left(p\right)}_{11}^{N} & L{\left(p\right)}_{12}^{N} & \cdots & L{\left(p\right)}_{1m}^{N}\\ L{\left(p\right)}_{21}^{N} & L{\left(p\right)}_{22}^{N} & \cdots & L{\left(p\right)}_{2m}^{N}\\ \vdots & \vdots & \ddots & \vdots \\ L{\left(p\right)}_{n1}^{N} & L{\left(p\right)}_{n2}^{N} & \cdots & L{\left(p\right)}_{nm}^{N} \end{array}\right]$$ (17)

In the index system presented in Section 2.3.1, there are some conflicting attribute indicators. For example, the worse the regime stability is, the greater the exchange rate change would be. That is, the attribute *c*_1_ and *c*_4_ are conflicting to some extents. To evaluate the FDI risk realistically, the evaluation method should take the conflicting characteristics of attributes into consideration. As mentioned above, the VIKOR method is an effective method for the MADM problems with conflicting attributes. It derives the compromise solution, which not only provides a maximum “group utility” for the “majority” but also minimize the “individual regret” for the “opponent”. Thus, the VIKOR method under probabilistic linguistic environment is presented to evaluate the FDI risk.

Up to now, the classical VIKOR method has been extended to many different situations and applied to solve different practical problems. However, most research are based on the quantitative information, such as fuzzy VIKOR [40], intuitionistic fuzzy VIKOR [41], and hesitant fuzzy VIKOR [42]. Some others are with linguistic information and different types of linguistic information representation forms have been studied, such as fuzzy linguistic VIKOR [43] and hesitant fuzzy linguistic VIKOR [34]. Zhang and Xing [44] studied the probabilistic linguistic VIKOR based on a probabilistic linguistic distance measure. No research develops a VIKOR-based method under the probabilistic linguistic environment to solve the FDI risk evaluation problem or FDI-related MADM problems.

As for the probabilistic linguistic VIKOR, choosing a proper measure function for *L_ρ_* −metric is crucial. The most used tool is distance measure. In Ref. [44], the probabilistic linguistic *L_ρ_* −metric is defined based on a probabilistic linguistic distance measure, which is extended by the idea of Hamming distance. However, there are many different distance measures, such as Hamming distance, Euclidean distance, and Hausdorff distance. No research on judging which one is best in different situations. To be more general, this paper proposes the generalized Hausdorff distance measure, which combines the idea of Hamming distance and Euclidean distance. Based on this, we present a PL-VIKOR method based on the new measure function to evaluate regional WHH degree. The details are presented as follows:

Firstly, the probabilistic linguistic positive ideal solution (PLPIS) and the probabilistic linguistic negative ideal solution (PLNIS) are defined as follows:

#### Definition 4 [14]

Given a normalized probabilistic linguistic decision matrix *ND* = (*L*(*p*)_*ij*_)*n*×*m* with *L*(*p*)_*ij*_ = {*L*^(*l*)^*p*^(*l*)^|*l* = 1,2,⋯,#*L*(*p*)}. The PLPIS and the PLNIS are presented respectively as:

$$L{\left(p\right)}^{+}={\left(L{\left(p\right)}_{1}^{+},L{\left(p\right)}_{2}^{+},\cdots ,L{\left(p\right)}_{m}^{+}\right)}^{T}$$ (18)

$$L{\left(p\right)}^{-}={\left(L{\left(p\right)}_{1}^{-},L{\left(p\right)}_{2}^{-},\cdots ,L{\left(p\right)}_{m}^{-}\right)}^{T}$$ (19)

where $L{\left(p\right)}_{j}^{+}=max_{i}L{\left(p\right)}_{ij}$ for the benefit-type attribute *c*_*j*_ and $L{\left(p\right)}_{j}^{-}=min_{i}L{\left(p\right)}_{ij}$ for the benefit-type attribute *c*_*j*_. As for the cost attributes, we can transfer them to the benefit-type. The max operator for the PLTS is introduced as follows:

1. If *L*(*p*)_1_ ≻ *L*(*p*)_2_, then *max*{*L*(*p*)_1_,*L*(*p*)_2_} = *L*(*p*)_1_, *min*{*L*(*p*)_1_,*L*(*p*)_2_} = *L*(*p*)_2_;
2. If *L*(*p*)_1_ ≺ *L*(*p*)_2_, then *max*{*L*(*p*)_1_,*L*(*p*)_2_} = *L*(*p*)_2_, *min*{*L*(*p*)_1_,*L*(*p*)_2_} = *L*(*p*)_1_.

Thus, the PLPIS and the PLNIS for the normalized probabilistic linguistic decision matrix can be determined as:

$$ND^{+}=\left\{max\;L{\left(p\right)}_{i1},max\;L{\left(p\right)}_{i2},\cdots ,max\;L{\left(p\right)}_{im}\right\}=\left\{L{\left(p\right)}_{1}^{+},L{\left(p\right)}_{2}^{+},\cdots ,L{\left(p\right)}_{m}^{+}\right\}$$ (20)

$$ND^{-}=\left\{min\;L{\left(p\right)}_{i1},min\;L{\left(p\right)}_{i2},\cdots ,min\;L{\left(p\right)}_{im}\right\}=\left\{L{\left(p\right)}_{1}^{-},L{\left(p\right)}_{2}^{-},\cdots ,L{\left(p\right)}_{m}^{-}\right\}$$ (21)

Inspired by the *L*_ρ_− metric of the classical VIKOR method as shown in Eq (2), the probabilistic linguistic *PLL_ρ_*−metric for the country *a*_*i*_ is introduced based on the PLPIS and the PLNIS:

$$PLL_{\rho ,i}={\left(\sum_{j=1}^{m}{\left(w_{j}\frac{d_{\text{Hau}}\left(L{\left(p\right)}_{j}^{+},L{\left(p\right)}_{ij}\right)}{d_{\text{Hau}}\left(L{\left(p\right)}_{j}^{+},L{\left(p\right)}_{j}^{-}\right)}\right)}^{\rho }\right)}^{\frac{1}{\rho }},0\leq \rho \leq \infty ;i=1,2,\cdots ,n$$ (22)

where *w*_*j*_ is the weight of the attribute *c*_*j*_, and satisfies 0 ≤ *w*_*j*_ ≤ 1, $\sum_{j=1}^{m}w_{j}=1$. $d_{Hau}\left(L{\left(p\right)}_{j}^{+},L{\left(p\right)}_{ij}\right)$ and $d_{Hau}\left(L{\left(p\right)}_{j}^{+},L{\left(p\right)}_{j}^{-}\right)$ are the generalized Hausdorff distance measures. The definition of the generalized Hausdorff distance measure is presented below:

#### Definition 5

Let $L{\left(p\right)}_{1}=\left\{L_{1}^{\left(l\right)}\left(p_{1}^{\left(l\right)}\right)|l=1,2,\cdots ,\#L{\left(p\right)}_{1}\right\}$ and $L{\left(p\right)}_{2}=\left\{L_{2}^{\left(l\right)}\left(p_{2}^{\left(l\right)}\right)|l=\right.\left.1,2,\cdots ,\#L{\left(p\right)}_{2}\right\}$ be two PLTSs based on the linguistic evaluation scale $S=\left\{s_{\alpha }|\alpha =-\tau ,\cdots ,-1,0,1,\cdots ,\tau \right\}$. The generalized Hausdorff distance between the PLTSs *L*(*p*)_1_ and *L*(*p*)_2_ can be defined as:

$$d_{Hau\;}\left(L{\left(p\right)}_{1},L{\left(p\right)}_{2}\right)=d_{Hau\;}\left({\left(L{\left(p\right)}_{1}\right)}^{N},{\left(L{\left(p\right)}_{2}\right)}^{N}\right)={\left(max_{l=1,2,\cdots ,\#L}{\left|\frac{p_{1}^{N(l)}\gamma_{1}^{N(l)}-p_{2}^{N(l)}\gamma_{2}^{N(l)}}{2\tau +1}\right|}^{\lambda }\right)}^{\frac{1}{\lambda }}$$ (23)

where *λ* > 0. Particularly, if *λ* = 0, then the generalized Hausdorff distance becomes the Hamming-Hausdorff distance:

$$d_{Hau\;}\left(L{\left(p\right)}_{1},L{\left(p\right)}_{2}\right)=max_{l=1,2,\cdots ,\#L}|\frac{p_{1}^{N(l)}\gamma_{1}^{N(l)}-p_{2}^{N(l)}\gamma_{2}^{N(l)}}{2\tau +1}|$$ (24)

If *λ* = 2, then the generalized Hausdorff distance becomes the Euclidean-Hausdorff distance:

$$d_{Hau\;}\left(L{\left(p\right)}_{1},L{\left(p\right)}_{2}\right)={\left(max_{l=1,2,\cdots ,\#L}{\left|\frac{p_{1}^{N(l)}\gamma_{1}^{N(l)}-p_{2}^{N(l)}\gamma_{2}^{N(l)}}{2\tau +1}\right|}^{2}\right)}^{\frac{1}{2}}$$ (25)

Based on the probabilistic linguistic *PLL*_*p*_−metric, the probabilistic linguistic group utility measure and the probabilistic linguistic individual regret measure of the country *a*_*i*_ are defined respectively as:

$$PLGU_{i}=PLL_{1,i}=\sum_{j=1}^{m}\left(w_{j}\frac{d_{Hau\;}\left(L{\left(p\right)}_{j}^{+},L{\left(p\right)}_{ij}\right)}{d_{Hau\;}\left(L{\left(p\right)}_{j}^{+},L{\left(p\right)}_{j}^{-}\right)}\right)$$ (26)

$$PLIR_{i}=PLL_{\infty ,i}=max\left(w_{j}\frac{d_{Hau}\left(L{\left(p\right)}_{j}^{+},L{\left(p\right)}_{ij}\right)}{d_{Hau\;}\left(L{\left(p\right)}_{j}^{+},L{\left(p\right)}_{j}^{-}\right)}\right)$$ (27)

where *w*_*j*_ is the weight of the attribute *c*_*j*_, and satisfies 0 ≤ *w*_*j*_ ≤ 1, $\sum_{j=1}^{m}w_{j}=1$. The probabilistic linguistic group utility measure is a separation measure between the country and the positive ideal solution. The smaller the value of *PLGU* is, the bigger the group utility should be. The probabilistic linguistic individual regret measure reflects the distance between the country and the negative ideal solution. The smaller the value of *PLIR*, the smaller the individual regret of the opponent.

Then, the probabilistic linguistic compromise measure of the country *a*_*i*_ is derived as:

$$PLC_{i}=\theta \frac{PLGU_{i}-PLGU^{min}}{PLGU^{\max}-PLGU^{min}+(1-\theta )\frac{PLIR_{i}-PLIR^{min}}{PLIR^{max^{min}}}}$$ (28)

where *PLGU*^max^ = *max*_*i*_{*PLGU*_*i*_}, $PLGU^{min\;min_{i}\left\{PLGU_{i}\right\}}$, $PLIR^{max\;\;max_{i}\left\{PLIR_{i}\right\}}$, $PLIR^{min\;\;min_{i}\left\{PLIR_{i}\right\}}$. *θ* is the attitudinal character parameter of the experts, which represents the weight of the strategy of (the majority of attribute or) the maximum group utility. 1-*θ* represents the weight factor for the individual regret. The value of *θ* is determined by the experts. When the experts have equal preference for the group utility and the individual regret, then let *θ* be 0.5; when the experts are more concerned about the group utility, then we have 0.5 < *θ* ≤ 1; when the experts are more concerned about the individual regret, then we have 0 ≤ *θ* < 0.5. The smaller the value of *PLC*_*i*_ is, the better the region *a*_*i*_ should be.

Finally, we rank the countries based on the values of *PLGU*_*i*_, *PLIR*_*i*_, and *PLC*_*i*_, respectively, three ranking lists are obtained. Then, the compromise solution deriving process is proposed as follows:

✓ The country $a_{1^{*}}$ would be a unique compromise solution, if it is ranked the first by the compromise measure *PLC*_*i*_ in ascending order, and satisfies the following two conditions:
 Condition 1) Acceptable advantage: $PLC_{a_{2^{*}}}-PLC_{a_{1^{*}}}\geq \frac{1}{n-1}$, where $a_{2^{*}}$ is the country in the second position in the ranking list by *PLC*_*i*_.
 Condition 2) Acceptable stability in decision making: the country $a_{1^{*}}$ should rank the first in the ranking list by *PLGU*_*i*_ and *PLIR*_*i*_.
✓ If these two conditions cannot be satisfied simultaneously, then more than one country is regarded as the compromise solutions:
  1. If Condition 2 is not satisfied, then the countries $a_{1^{*}}$ and $a_{2^{*}}$ make up the set of the compromise solutions.
  2. If Condition 1 is not satisfied, then the countries $a_{1^{*}},a_{2^{*}},\cdots ,a_{n^{*}}$ make up the set of the compromise solutions, where $a_{n^{*}}$ is determined by $PLC_{a_{n^{*}}}-PLC_{a_{1^{*}}}<\frac{1}{\left(n-1\right)}$ for the maximum n^*^ (the position of these countries are “in closeness”).

In addition, the whole evaluation process is summarized in the following algorithm:

**Algorithm 1**

**Input:** The index system and the assessment values of countries concerning each attribute.
**Output**: The compromise solution.
**Step 1.** Establish the index system for evaluating the FDI risk.
**Step 2.** Construct the probabilistic linguistic decision matrix *D* = (*L*(*p*)_*ij*_)*n*×*m* and then normalize it into $ND={\left(L{\left(p\right)}_{ij}^{N}\right)}_{n\times m}$.
**Step 3.** Determine the subject attribute weights by consulting the experts, calculate the object attribute weights by using Eq (14), and determine the final attribute weights by using Eq (15).
**Step 4.** Determine PLPIS $ND^{+}=\left\{L{\left(p\right)}_{1}^{+},L{\left(p\right)}_{2}^{+},\cdots ,L{\left(p\right)}_{m}^{+}\right\}$ and PLNIS $ND^{-}=\left\{L{\left(p\right)}_{1}^{-},L{\left(p\right)}_{2}^{-},\cdots ,L{\left(p\right)}_{m}^{-}\right\}$ for the normalized probabilistic linguistic decision matrix by using Eqs (20) and (21), and calculate the generalized Hausdorff distance between the PLTSs $L{\left(p\right)}_{j}^{+}$ and $L{\left(p\right)}_{ij}$ by using Eq (23). Then, the probabilistic linguistic group utility measures *PLGU*_*i*_ (*i* = 1,2,⋯,*n*) and the probabilistic linguistic individual regret measures $PLIR_{i}$ (*i* = 1,2,⋯,*n*) can be calculated according to Eqs (26) and (27).
**Step 5.** Use Eq (28) to calculate the probabilistic linguistic compromise measures *PLC*_*i*_ (*i* = 1,2,⋯,*n*)
**Step 6**. Rank the countries *a*_*i*_ (*i* = 1,2,⋯,*n*) based on the values of *PLGU*_*i*_, *PLIR*_*i*_, and *PLC*_*i*_, respectively, and obtain three ranking lists.
**Step 7.** Derive the compromise solution according to the compromise solution deriving process mentioned above.
**Step 8.** End.

**Fig 2:**
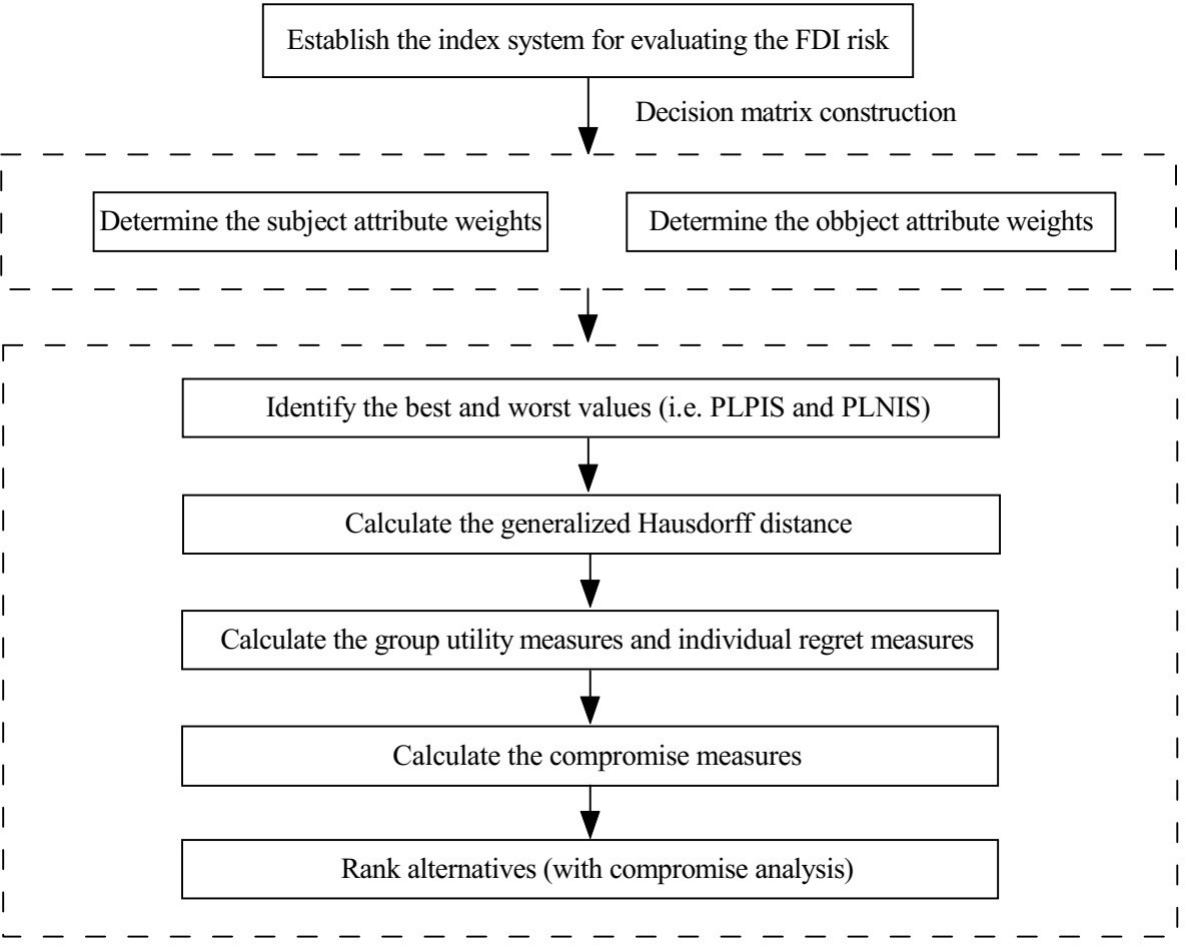
General framework for FDI risk evaluation.

The whole evaluation framework can be presented in the following Fig 2.

## 3. Case study

In this section, an example is illustrated to show the proposed method for FDI risk evaluation. As Asian region is the most popular for international investors, its FDI has increased for the third consecutive year during the pandemic of COVID-19, reaching a new historical high. Here, we take into consideration the four host countries, Singapore (*a*_1_), Brunei (*a*_2_), Indonesia (*a*_3_), and Philippines (*a*_4_). Based on the linguistic evaluation scale *S* = {*s*_-3_,*s*_-2_,*s*_-1_,*s*_0_,*s*_1_.*s*_2_,*s*_3_} (*s*_-3_ = *verypoor*, *s*_-2_ = *poor*, *s*_-1_ = *alittlepoor*, *s*_0_ = *middling*, *s*_1_ = *alittlegood*, *s*_2_ = *good*, *s*_3_ = *verygood*), ten experts provide their judgments with linguistic terms. Then, we collect all the judgments of the experts and get the final assessment values in the form of the PLTSs. For example, when the experts are evaluating the performance of the country *a*_1_ concerning the attribute *c*_1_, four experts think that it is “*middling* (*s*_0_)”, six experts think that it is “*alittlegood* (*s*_1_)”. Thus, the assessment value of the country *a*_1_ concerning the attribute *c*_1_ is represented in the form of the PLTS, *L*(*p*)_11_ = {*s*_0_(0.4),*s*_1_(0.6)}. What’s more, when the experts are evaluating the performance of the country *a*_3_ concerning the attribute *c*_1_, six expert thinks that it is “*middling* (*s*_0_)”, two experts think that it is “*alittlegood* (*s*_1_)”, and the other one expert cannot give his/her judgment because of his/her limited knowledge and cognition. Thus, the final assessment value of the country *a*_3_ concerning the attribute *c*_1_ is obtained in the form of the PLTS, *L*(*p*)_31_ = {*s*_0_(0.6),*s*_1_(0.2)}. Similarly, we collect all the probabilistic linguistic judgments and construct a probabilistic linguistic decision matrix, presented in Table 2. It should be noted that, in Table 2, all the assessment values of cost-attribute indicators have been processed using the transformation function mentioned in Section 2.3.3.

**Table 2:** The probabilistic linguistic decision matrix.

	*c*_1_	*c*_2_	*c*_3_	*c*_4_
*a*_1_	{*s*_0_(0.4),*s*_1_(0.6)}	{*s*_-1_(0.2),*s*_1_(0.8)}	{*s*_0_(0.2),*s*_1_(0.4)}	{*s*_0_(0.4),*s*_2_(0.6)}
*a*_2_	{*s*_2_(0.2),*s*_0_(0.8)}	{*s*_-1_(0.4),*s*_0_(0.6)}	{*s*_0_(0.6),*s*_1_(0.2)}	{*s*_2_(0.8)}
*a*_3_	{*s*_0_(0.6),*s*_1_(0.2)}	{*s*_3_(0.8)}	{*s*_-2_(0.2),*s*_0_(0.4),*s*_1_(0.2)}	{*s*_1_(0.8),*s*_3_(0.2)}
*a*_4_	{*s*_-1_(0.4),*s*_0_(0.4)}	{*s*_-2_(0.2),*s*_0_(0.6)}	{*s*_-1_(0.2),*s*_0_(0.8)}	{*s*_-1_(0.4),*s*_1_(0.4)}
	*c*_5_	*c*_6_	*c*_7_	*c*_8_
*a*_1_	{*s*_1_(0.2),*s*_-1_(0.8)}	{*s*_-1_(0.2),*s*_-1_(0.4),*s*_1_(0.2)}	{*s*_1_(0.6),*s*_2_(0.2)}	{*s*_0_(0.2),*s*_-1_(0.8)}
*a*_2_	{*s*_-2_(0.8)}	{*s*_0_(0.4),*s*_1_(0.4),}	{*s*_-2_(0.2),*s*_-1_(0.8)}	{*s*_0_(0.4),*s*_1_(0.6)}
*a*_3_	{*s*_-1_(0.2),*s*_0_(0.4)}	{*s*_-1_(0.6),*s*_2_(0.2)}	{*s*_-1_(0.2),*s*_1_(0.4)}	{*s*_2_(0.4),*s*_3_(0.4)}
*a*_4_	{*s*_1_(0.2),*s*_2_(0.6)}	{*s*_0_(0.8),*s*_2_(0.2)}	{*s*_0_(0.6),*s*_1_(0.4)}	{*s*_1_(0.8)}

After normalization and conducting the ranking rule, the normalized probabilistic linguistic decision matrix in ascending order with the same length is obtained, as shown in Table 3.

**Table 3:** The normalized probabilistic linguistic decision matrix in ascending order.

	*c*_1_	*c*_2_	*c*_3_	*c*_4_
*a*_1_	{*s*_0_(0),*s*_0_(0.4),*s*_1_(0.6)}	{*s*_-1_(0.2),*s*_-1_(0),*s*_1_(0.8)}	{*s*_0_(0),*s*_0_(0.33),*s*_1_(0.67)}	{*s*_0_(0),*s*_0_(0.4),*s*_2_(0.6)}
*a*_2_	{*s*_0_(0),*s*_0_(0.8),*s*_2_(0.2)}	{*s*_-1_(0.4),*s*_-1_(0),*s*_0_(0.6)}	{*s*_0_(0),*s*_0_(0.75),*s*_1_(0.25)}	{*s*_2_(0),*s*_2_(0),*s*_2_(1)}
*a*_3_	{*s*_0_(0),*s*_0_(0.75),*s*_1_(0.25)}	{*s*_3_(0),*s*_3_(0),*s*_3_(1)}	{*s*_-2_(0.25),*s*_0_(0.5),*s*_1_(0.25)}	{*s*_1_(0),*s*_3_(0.2),*s*_1_(0.8)}
*a*_4_	{*s*_-1_(0.5),*s*_-1_(0),*s*_0_(0.5)}	{*s*_-2_(0.25),*s*_-2_(0),*s*_0_(0.75)}	{*s*_-1_(0.2),*s*_-1_(0),*s*_0_(0.8)}	{*s*_-1_(0.5),*s*_-1_(0),*s*_1_(0.5)}
	*c*_5_	*c*_6_	*c*_7_	*c*_8_
*a*_1_	{*s-*_1_(0.8),*s-*_1_(0),*s*_1_(0.2)}	{*s-*_2_(0.25),*s-*_1_(0.5),*s*_1_(0.25)}	{*s*_1_(0),*s*_2_(0.25),*s*_1_(0.75)}	{*s*_-1_(0.8),*s*_-1_(0),*s*_0_(0.2)}
*a*_2_	{*s*_-2_(1),*s*_-2_(0),*s*_-2_(0)}	{*s*_0_(0),*s*_0_(0.5),*s*_1_(0.5)}	{*s*_-1_(0.8),*s*_-2_(0.2),*s*_-2_(0)}	{*s*_0_(0),*s*_0_(0.4),*s*_1_(0.6)}
*a*_3_	{*s*_-1_(0.6),*s*_-1_(0),*s*_0_(0.4)}	{*s*_-1_(0.75),*s*_-1_(0),*s*_2_(0.25)}	{*s*_-1_(0.33),*s*_-1_(0),*s*_1_(0.67)}	{*s*_2_(0),*s*_2_(0.5),*s*_3_(0.5)}
*a*_4_	{*s*_1_(0),*s*_1_(0.25),*s*_2_(0.75)}	{*s*_0_(0),*s*_0_(0.8),*s*_2_(0.2)}	{*s*_0_(0),*s*_0_(0.6),*s*_1_(0.4)}	{*s*_1_(0),*s*_1_(0),*s*_1_(1)}

The subjective weights $\varpi_{j}$ (*j* = 1,2,⋯,*m*) are provided by the experts as $\varpi_{1}$ = 0.15, $\varpi_{2}$ = 0.15, $\varpi_{3}$ = 0.15, $\varpi_{4}$ = 0.1, $\varpi_{5}$ = 0.2, $\varpi_{6}$ = 0.1, $\varpi_{7}$ = 0.1, and $\varpi_{8}$ = 0.05. Without loss of generality, let *q* = 1 and ε = 0.5, then the objective weights *ω*_*j*_ (*j* = 1,2,⋯,*m*) are obtained according to Eq (14): *ω*_1_ = 0.082, *ω*_2_ = 0.110, *ω*_3_ = 0.078, *ω*_4_ = 0.186, *ω*_5_ = 0.072, *ω*_6_ = 0.12, *ω*_7_ = 0.134, *ω*_8_ = 0.218, and the final weight vector of the attributes is obtained according to Eq (15): $W={\left(w_{1},w_{2},\cdots ,w_{8}\right)}^{T}={\left(0.116,0.130,0.114,0.143,0.136,0.110,0.117,0.134\right)}^{T}$

The positive ideal solution and the negative ideal solution are presented in Table 4.

**Table 4:** The probabilistic linguistic ideal solutions.

	*c*_1_	*c*_2_	*c*_3_	*c*_4_
$L{\left(p\right)}_{j}^{+}$	{*s*_0_(0),*s*_0_(0.4),*s*_1_(0.6)}	{*s*_3_(0),*s*_3_(0),*s*_3_(1)}	{*s*_0_(0),*s*_0_(0.33),*s*_1_(0.67)}	{*s*_2_(0),*s*_2_(0),*s*_2_(1)}
$L{\left(p\right)}_{j}^{-}$	{*s*_-1_(0.5),*s*_-1_(0),*s*_0_(0.5)}	{*s*_-1_(0.4),*s*_-1_(0),*s*_0_(0.6)}	{*s*_-2_(0.25),*s*_0_(0.5),*s*_1_(0.25)}	{*s*_-1_(0.5),*s*_-1_(0),*s*_1_(0.5)}
	*c*_5_	*c*_6_	*c*_7_	*c*_8_
$L{\left(p\right)}_{j}^{+}$	{*s*_1_(0),*s*_-1_(0.25),*s*_2_(0.75)}	{*s*_0_(0),*s*_0_(0.5),*s*_1_(0.5)}	{*s*_1_(0),*s*_2_(0.25),*s*_1_(0.75)}	{*s*_2_(0),*s*_2_(0.5),*s*_3_(0.5)}
$L{\left(p\right)}_{j}^{-}$	{*s*_-2_(1),*s*_-2_(0),*s*_-2_(0)}	{*s*_-2_(0.25),*s*_-1_(0.5),*s*_1_(0.25)}	{*s*_*-*1_(0.8),*s*_*-*2_(0.2),*s*_-2_(0)}	{*s*_-1_(0.8),*s*_-1_(0),*s*_0_(0.2)}

Without loss of generality, let *λ* = 1 and we get the distances between the PLTSs (See Table 5). Then, the experts give the value of the parameter *θ* as *θ* = 0.5 and the values of *PLGU*_*i*_, *PLIR*_*i*_, and *PLC*_*i*_ are calculated according to Eqs (26)-(28), as shown in Table 6.

**Table 5:** The distance between the PLTSs.

	*c*_1_	*c*_2_	*c*_3_	*c*_4_
$d\left(L{\left(p\right)}_{j}^{+},L{\left(p\right)}_{j}^{-}\right)$	0.086	0.429	0.096	0.214
$d\left(L{\left(p\right)}_{j}^{+},L{\left(p\right)}_{1j}\right)$	0	0.314	0	0.114
$d\left(L{\left(p\right)}_{j}^{+},L{\left(p\right)}_{2j}\right)$	0.029	0.429	0.060	0
$d\left(L{\left(p\right)}_{j}^{+},L{\left(p\right)}_{3j}\right)$	0.050	0	0.071	0.171
$d\left(L{\left(p\right)}_{j}^{+},L{\left(p\right)}_{4j}\right)$	0.086	0.429	0.096	0.214
	*c*_5_	*c*_6_	*c*_7_	*c*_8_
$d\left(L{\left(p\right)}_{j}^{+},L{\left(p\right)}_{j}^{-}\right)$	1	0.107	0.129	0.214
$d\left(L{\left(p\right)}_{j}^{+},L{\left(p\right)}_{1j}\right)$	0.829	0.071	0	0.214
$d\left(L{\left(p\right)}_{j}^{+},L{\left(p\right)}_{2j}\right)$	1	0	0.129	0.143
$d\left(L{\left(p\right)}_{j}^{+},L{\left(p\right)}_{3j}\right)$	0.800	0.107	0.071	0
$d\left(L{\left(p\right)}_{j}^{+},L{\left(p\right)}_{4j}\right)$	0	0.014	0.071	0.143

**Table 6:** The values of *PLGU*_*i*_, *PLIR*_*i*_, and *PLC*_*i*_.

Region	*PLGU*_*i*_	*PLIR*_*i*_	*PLC*_*i*_
*a*_1_	0.528	0.134	0.110
*a*_2_	0.582	0.136	0.444
*a*_3_	0.607	0.166	0.822
*a*_4_	0.680	0.143	0.722

Based on Table 6, *PLGU*_*i*_, *PLIR*_*i*_, and *PLC*_*i*_ are ranked in ascending order, respectively:

$$PLGU_{1}<PPLGU_{2}<PLGU_{3}<LGU_{4}$$

$$PLIR_{1}<\;PLIR_{2}<\;PLIR_{4}<\;PLIR_{3}$$

$$PLC_{1}<PLC_{2}<PLC_{4}<PLC_{3}$$

The country *a*_1_ has the minimal values of *PLGU*, *PLIR*, and *PLC*. Meanwhile, $PLC_{2}-PLC_{1}=0.334>\frac{1}{\left(n-1\right)}=\frac{1}{3}=0.33$. Thus, *a*_1_ is the unique compromise solution. That is, *a*_1_ is the optimal choice for FDI.

## 4. Results

### 4.1. Sensitivity analysis: Results based on the different values of the parameters

1. Results based on the different values of the parameter *θ*

A sensitivity analysis by changing the values of the parameter *θ* is conducted concerning the case study. The ranking results of the four candidate countries with the different values of the parameter *θ* are represented in Table 7. It is obvious that when the values of the parameter *θ* are changed, the ranking results are unchanged. Overall, the ranking results are almost *a*_1_ < *a*_2_ < *a*_4_ < *a*_3_ based on *PLGU*, *PLIR*, and *PLC*.

**Table 7:** The ranking results based on the different values of *θ*.

*θ*	*PLC*_1_	*PLC*_2_	*PLC*_3_	*PLC*_4_	Ranking	Compromise solution
0	0.142	0.171	0.850	0.453	*PLC*_1_<*PLC*_2_<*PLC*_4_<*PLC*_3_	*a*_1_,*a*_2_,*a*_4_
0.1	0.136	0.239	0.844	0.507	*PLC*_1_<*PLC*_2_<*PLC*_4_<*PLC*_3_	*a*_1_,*a*_2_
0.2	0.130	0.298	0.838	0.560	*PLC*_1_<*PLC*_2_<*PLC*_4_<*PLC*_3_	*a*_1_,*a*_2_
0.3	0.123	0.308	0.833	0.614	*PLC*_1_<*PLC*_2_<*PLC*_4_<*PLC*_3_	*a*_1_,*a*_2_
0.4	0.117	0.376	0.827	0.668	*PLC*_1_<*PLC*_2_<*PLC*_4_<*PLC*_3_	*a*_1_,*a*_2_
0.5	0.110	0.444	0.822	0.722	*PLC*_1_<*PLC*_2_<*PLC*_4_<*PLC*_3_	*a*_1_
0.6	0.104	0.512	0.816	0.776	*PLC*_1_<*PLC*_2_<*PLC*_3_<*PLC*_4_	*a*_1_
0.7	0.097	0.580	0.810	0.830	*PLC*_1_<*PLC*_2_<*PLC*_3_<*PLC*_4_	*a*_1_
0.8	0.091	0.648	0.804	0.883	*PLC*_1_<*PLC*_2_<*PLC*_3_<*PLC*_4_	*a*_1_
0.9	0.084	0.717	0.799	0.937	*PLC*_1_<*PLC*_2_<*PLC*_3_<*PLC*_4_	*a*_1_
1	0.078	0.785	0.793	0.991	*PLC*_1_<*PLC*_2_<*PLC*_3_<*PLC*_4_	*a*_1_

From Table 7, we know that:

- When the parameter *θ* is equal to 0, *Condition 1* is not satisfied. The countries *a*_1_, *a*_2_, and *a*_4_ are the compromise solutions. That is, the countries *a*_1_, *a*_2_, and *a*_4_ have the best performance for FDI.
- When the parameter *θ* assigns 0, 0.1, 0.2, 0.3, and 0.4, respectively, *Condition 1* is not satisfied. The countries *a*_1_ and *a*_2_ are the compromise solutions. That is, *a*_1_ and *a*_2_ have the best performance for FDI.
- When the parameter *θ* equals to 0.5, 0.6, 0.7, 0.8, 0.9, and 1, respectively, the country *a*_1_ is the optimal solution. That is, *a*_1_ has the best performance for FDI.
- When the parameter *θ* increases, the values of the *PLC*_*i*_ for four candidate countries have the same change rate.
- When the value of the parameter *θ* changes, the best country for FDI is different.
  2. Results based on the different values of the parameter *λ*

The probabilistic linguistic group utility measure and the probabilistic linguistic individual regret measure are based on the Hausdorff distance measure, which is determined based on the parameter *λ*. Thus, it is necessary to make a sensitivity analysis by changing the parameter *λ*. Let the parameter *λ* take different values, such as 1, 2, 3, 4 and 5, respectively. Then through calculating we know that the distances between the PLTSs do not change with the different values of the parameter *λ* (See Table 8). Thus, the ranking results of the four countries with the different values of the parameter *λ* are the same.

**Table 8:** The distance between the PLTSs when the parameters *λ* = 1,2,3,4,5, respectively.

Distance	*c*_1_	*c*_2_	*c*_3_	*c*_4_
$d\left(L{\left(p\right)}_{j}^{+},L{\left(p\right)}_{j}^{-}\right)$	0.086	0.429	0.096	0.214
$d\left(L{\left(p\right)}_{j}^{+},L{\left(p\right)}_{1j}\right)$	0	0.314	0	0.114
$d\left(L{\left(p\right)}_{j}^{+},L{\left(p\right)}_{2j}\right)$	0.029	0.429	0.060	0
$d\left(L{\left(p\right)}_{j}^{+},L{\left(p\right)}_{3j}\right)$	0.050	0	0.071	0.171
$d\left(L{\left(p\right)}_{j}^{+},L{\left(p\right)}_{4j}\right)$	0.086	0.429	0.096	0.214
	*c*_5_	_c6_	*c*_7_	*c*_8_
$d\left(L{\left(p\right)}_{j}^{+},L{\left(p\right)}_{j}^{-}\right)$	1	0.107	0.129	0.214
$d\left(L{\left(p\right)}_{j}^{+},L{\left(p\right)}_{1j}\right)$	0.829	0.071	0	0.214
$d\left(L{\left(p\right)}_{j}^{+},L{\left(p\right)}_{2j}\right)$	1	0	0.129	0.143
$d\left(L{\left(p\right)}_{j}^{+},L{\left(p\right)}_{3j}\right)$	0.800	0.107	0.071	0
$d\left(L{\left(p\right)}_{j}^{+},L{\left(p\right)}_{4j}\right)$	0	0.014	0.071	0.143

### 4.2. Compared with other methods

In terms of the research on investment risk assessment of FDI, many are focusing on the risk assessment system, and mainly have the similar risk indicator, i.e., economics and finance, politics, environment, society and culture, law and regulation. While the studies use many kinds of research methods to evaluate the investment risk, and mainly from the perspective of fuzzy mathematics.

To illustrate the effectiveness of the proposed method, we make some comparative analysis with the other MADM methods concerning the above case study.

1. Compared with the PL-TOPSIS method

TOPSIS (Technique for Order Preference by Similarity to an Ideal Solution) method is a kind of commonly used effective MADM method. To verify the validity of our approach, the PL-VIKOR method is firstly compared with the TOPSIS method based on the PLTSs (denoted as the PL-TOPSIS method). Firstly, the PLPIS $L{\left(p\right)}^{+}=\left(L{\left(p\right)}_{1}^{+},L{\left(p\right)}_{2}^{+},\cdots ,L{\left(p\right)}_{m}^{+}\right)$ and the PLNIS $L{\left(p\right)}^{-}=\left(L{\left(p\right)}_{1}^{-},L{\left(p\right)}_{2}^{-},\cdots ,L{\left(p\right)}_{m}^{-}\right)$ should be determined. In the previous section, the PLPIS *L*(*p*)^+^ and the PLNIS *L*(*p*)^-^ have been presented in Table 4. Based on the attribute weights and the ideal solutions, the following steps of the PL-TOPSIS [14] are conducted.

We calculate the deviation degrees between each country and the PLPIS/PLNIS by

$$\begin{array}{r} dev\left(a_{i},L{\left(p\right)}^{+}\right)=\sum_{j=1}^{m}\left(w_{j}dev\left(L{\left(p\right)}_{ij},L{\left(p\right)}_{j}^{+}\right)\right)=\\ \sum_{j=1}^{m}w_{j}\sqrt{\frac{1}{\#L{\left(p\right)}_{ij}}\sum_{k=1}^{\#L{\left(p\right)}_{ij}}{\left(p_{ij}^{\left(k\right)}\gamma_{ij}^{\left(k\right)}-p_{j}^{+(k)}\gamma_{j}^{+(k)}\right)}^{2}} \end{array}$$ (29)

$$\begin{array}{c} dev\left(a_{i},L{\left(p\right)}^{-}\right)=\sum_{j=1}^{m}\left(w_{j}dev\left(L{\left(p\right)}_{ij},L{\left(p\right)}_{j}^{-}\right)\right)=\\ \sum_{j=1}^{m}w_{j}\sqrt{\frac{1}{\#L{\left(p\right)}_{ij}}\sum_{k=1}^{\#L{\left(p\right)}_{ij}}{\left(p_{ij}^{\left(k\right)}\gamma_{ij}^{\left(k\right)}-p_{j}^{-(k)}\gamma_{j}^{-(k)}\right)}^{2}} \end{array}$$ (30)

Let *dev*(*a*_*i*_,*L*(*p*)^+^)*min*_*i*_ *d* (*a*_*i*_,*L*(*p*)^+^)_*min*_ be the smallest deviation degree between the country *a*_*i*_ and the PLPIS, and *dev*(*a*_*i*_,*L*(*p*)^-^)*max*_*i*_ *d* (*a*_*i*_,*L*(*p*)-)_*max*_ be the largest deviation degree between the country *a*_*i*_ and the PLNIS. The closeness coefficients of different countries are calculated to rank them:

$$CI_{i}=\frac{dev\left(a_{i},L{\left(p\right)}^{-}\right)}{dev{\left(a_{i},L{\left(p\right)}^{-}\right)}_{max}\frac{dev\left(a_{i},L{\left(p\right)}^{+}\right)}{dev{\left(a_{i},L{\left(p\right)}^{+}\right)}_{min}}}$$ (31)

The larger the closeness coefficient is, the better the country should be. That is to say, the country with the largest closeness coefficient is the best choice for investment. In this way, the results by conducting the PL-TOPSIS method are derived and presented in Table 9.

**Table 9:** Closeness coefficients.

	*dev* ^+^	*dev* ^-^	*CI*_*i*_	Ranking
*a*_1_	0.108	0.210	0.659	2
*a*_2_	0.116	0.225	0.662	1
*a*_3_	0.217	0.114	0.344	4
*a*_4_	0.199	0.120	0.377	3

From Table 9, *a*_2_ is the country which has the best performance for FDI. When the parameter *θ* ≤ 0.5, the rankings of the countries *a*_3_ and *a*_4_ are consistent with the results obtained by the PL-TOPSIS method; however, the rankings of the countries *a*_1_ and *a*_2_ are changed. When the parameter 0.5 < *θ* ≤1, the rankings of all the countries have changed. The best choice obtained by the PL-TOPSIS method is different from the best choice obtained by the PL-VIKOR method, no matter how the parameter *θ* changes. The PL-TOPSIS method only considers the distances of the countries from the positive ideal solution and from the negative ideal solution. It does not consider the relative importance degree between the positive ideal solution and the negative ideal solution. Meanwhile, the solution derived from the PL-TOPSIS method is not always the closest to the ideal solution. Thus, the results derived from the PL-VIKOR method is more reasonable than the ones derived from PL-TOPSIS method.

1. Compared with the aggregation-based method

We can also directly use the aggregation operators to integrate the decision information and solve the case. Some aggregation operators of the PLTSs were introduced in Ref. [14]. For example, the probabilistic linguistic weighted averaging (PLWA) operator is defined as:

$$\begin{array}{l} PLWA\left(L{\left(p\right)}_{1},L{\left(p\right)}_{2},\cdots ,L{\left(p\right)}_{n}\right)=\cup_{L_{1}^{\left(l\right)}\in L{\left(p\right)}_{1}}\left\{w_{1}p_{1}^{\left(l\right)}L_{1}^{\left(l\right)}\right\}⊕\cup_{L_{2}^{\left(l\right)}\in L_{2}(p)}\left\{w_{2}p_{2}^{\left(l\right)}L_{2}^{\left(l\right)}\right\}⊕\cdots \\ ⊕\cup_{L_{n}^{\left(l\right)}\in L{\left(p\right)}_{n}}\left\{w_{n}p_{n}^{\left(l\right)}L_{n}^{\left(l\right)}\right\} \end{array}$$ (32)

The probabilistic linguistic weighted geometric (PLWG) operator is defined as:

$$\begin{array}{l} PLWG\left(L{\left(p\right)}_{1},L{\left(p\right)}_{2},\cdots ,L{\left(p\right)}_{n}\right)\\ ={\left(L{\left(p\right)}_{1}\right)}^{w_{1}}⊗{\left(L{\left(p\right)}_{2}\right)}^{w_{2}}⊗\cdots ⊗{\left(L{\left(p\right)}_{n}\right)}^{w_{n}}\\ =\cup_{L_{1}^{\left(l\right)}\in L{\left(p\right)}_{1}}\left\{{\left(L_{1}^{\left(l\right)}\right)}^{w_{1}p_{1}^{\left(l\right)}}\right\}⊗\cup_{L_{2}^{\left(l\right)}\in L{\left(p\right)}_{2}}\left\{{\left(L_{2}^{\left(l\right)}\right)}^{w_{2}p_{2}^{\left(l\right)}}\right\}⊗\cdots ⊗\cup_{L_{n}^{\left(l\right)}\in L{\left(p\right)}_{n}}\left\{{\left(L_{n}^{\left(l\right)}\right)}^{w_{n}p_{n}^{\left(l\right)}}\right\} \end{array}$$ (33)

By using the PLWA operator and the PLWG operator to directly integrate the decision information respectively, we obtain the decision results in Table 10.

**Table 10:** The results by using the aggregation operators.

Aggregation operator	Result
PLWA operator	*a*_2_ > *a*_1_ > *a*_3_ > *a*_4_
PLWG operator	*a*_2_ > *a*_1_ > *a*_3_ > *a*_4_

Table 10 shows that *a*_2_ is the country with the best performance for FDI. The results are different from the result by using the PL-VIKOR method. The PLWA operator is based on the arithmetic mean and the PLWG operator is based on the geometric mean. However, in real-life decision-making problem, it is usually difficult to capture the interrelationship among the decision information by using the aggregation operators. Furthermore, when using the aggregation operators to directly integrate the decision information, some useful and original information might be lost. This would lead to unreasonable solutions for the decision-making problems. Thus, our method is more rational than the aggregation-based methods.

## 5. Further discussion

Based on the analysis presented above, the PL-VIKOR method possesses the following notable advantages:

1. The PL-VIKOR method is proposed to handle the FDI risk evaluation problems in which the judgments of the countries with respect to the attributes are expressed by linguistic terms. The linguistic terms are in accordance with human’s way of thinking and suitable for expressing uncertain decision-making information. However, some extended VIKOR methods in Refs. [35–37] cannot be used to solve the FDI risk evaluation problems with linguistic information. The PL-VIKOR method can solve this problem exactly. Thus, the PL-VIKOR method is more powerful.
2. The PL-VIKOR method is based on the behavior preference parameter *θ*. The parameter *θ* helps the experts to obtain the compromise solution by taking the appropriate value of *θ* based on the experts’ preferences. When the experts have equal preference for the group utility and the individual regret, then let *θ* be 0.5; when the experts are more concerned about the group utility, then we have 0.5 < *θ* ≤ 1; when the experts are more concerned about the individual regret, then we have 0 ≤ *θ* < 0.5. Thus, the proposed PL-VIKOR method is more flexible and can effectively improve the decision reliability.
3. Compared with the methods in Refs. [34, 43, 44]: a) The method in Ref. [34] is based on the hesitant fuzzy linguistic information, the method in Ref. [43] is based on the traditional fuzzy linguistic information, and the method in Ref. [44] and our approach are based on the probabilistic linguistic information. We have stated that the experts are more inclined to use PLTSs to express their assessment values and it is necessary to evaluate the FDI risk under the probabilistic linguistic environment. The methods in Refs. [34, 44] cannot solve the FDI risk evaluation problem with PLTSs. b) The method in Ref. [44] and our approach both study the VIKOR method with PLTSs. However, the method in Ref. [44] is developed for green supply chain initiatives evaluation with main-criteria and sub-criteria and our method is developed for the FDI risk evaluation. Moreover, the proposed PL-VIKOR method is based on the generalized measure function and thus is more reasonable and general than the method in Ref. [44].
4. Compared with the PL-TOPSIS method: The PL-VIKOR method derives the compromise solution, which not only maximizes the group utility for the majority but also minimizes the individual regret for the opponent. However, The PL-TOPSIS method only considers the distance of each country from the positive ideal solution and from the negative ideal solution. It does not consider the relative importance degrees of the countries between the positive ideal solution and the negative ideal solution. This means that the solution derived from the PL-TOPSIS method is not always the closest one to the ideal solution. While the compromise solution derived from the PL-VIKOR method is the closest to the ideal solution.
5. Compared with the aggregation-based method: The PL-VIKOR method is based on the closeness to the ideal solution. In real-life decision-making problems, it is usually difficult to capture the interrelationship of the decision-making information. The aggregation-based method, which uses aggregation operators to directly integrate the decision-making information, may lose some useful and original information. This would lead to unreasonable solutions for the decision-making problem. The proposed PL-VIKOR method is more convincing and rational for the MADM problem with PLTSs.

It should be noted that the PL-VIKOR method also has some disadvantages: (1) It is only applicable for the MADM problem when attributes conflict in a probabilistic linguistic environment. (2) Decision makers dealing with conflict need to be able to accept compromise solutions. If decision maker cannot accept compromise solutions, then the solution obtained by this method is not satisfactory to DMs. (3) The measurement function is important. Therefore, how to select reasonable and suitable measurement functions is critical. Different measurement functions may derive different results.

## 6. Conclusions

Considering the inherent uncertainties, fuzziness and the conflicts within certain attributes, this paper presents a novel approach for evaluating FDI risk through the development of a probabilistic linguistic method. The initial step involves constructing an index system comprising eight indicators derived from the underlying associated with FDI risk. Secondly, we have obtained the attribute weights by integrating subjective attribute weight information (i.e., experts’ knowledge) with objective attribute weight information. The objective attribute weights are computed using a programming model that utilizes probabilistic linguistic entropy and cross entropy. Thirdly, the FDI risk evaluation method has been proposed utilizing the PL-VIKOR method in conjunction with a novel measurement function. Furthermore, the developed methodology has been implemented in a practical case study, providing valuable insights. Through comparative analysis, we have identified several key associated with our approach. Specifically, our method excels in accurately representing the primary decision-making information and effectively addressing the impact of conflicting attributes on decision outcomes. Consequently, our approach aligns more closely with real-world scenarios, enhancing its applicability and efficacy.
